# Whole-genomic comparison reveals complex population dynamics and parasitic adaptation of *Echinococcus granulosus sensu stricto*

**DOI:** 10.1128/mbio.03256-24

**Published:** 2025-04-10

**Authors:** Yao-Dong Wu, Zirui Ren, Li Li, Wen-Hui Li, Nian-Zhang Zhang, Yan-Tao Wu, Guo-Dong Dai, Wei-Gang Chen, Wen-Jie Mu, Shuai Wang, Jiandong Li, Qin Yu, Xue-Peng Cai, Xin Jin, Bao-Quan Fu, Daxi Wang, Wan-Zhong Jia, Hong-Bin Yan

**Affiliations:** 1 State Key Laboratory for Animal Disease Control and Prevention/College of Veterinary Medicine, Lanzhou University/National Para-reference Laboratory for Animal Echinococcosis/Gansu Province Research Center for Basic Disciplines of Pathogen Biology/Key Laboratory of Veterinary Parasitology of Gansu Province/Key Laboratory of Veterinary Etiological Biology and Key Laboratory of Ruminant Disease Prevention and Control (West), Ministry of Agricultural and Rural Affairs/Lanzhou Veterinary Research Institute, Chinese Academy of Agricultural Sciences 12661, Lanzhou, China; 2 BGI Research 213636 , Beijing, China; 3 Shenzhen Key Laboratory of Unknown Pathogen Identification, BGI Research 213636 , Shenzhen, China; 4 Jiangsu Co‑Innovation Center for Prevention and Control of Important Animal Infectious Diseases and Zoonoses, Yangzhou, China; University of Cambridge, Cambridge, United Kingdom; University of Glasgow, Glasgow, United Kingdom

**Keywords:** *Echinococcus granulosus sensu stricto*, population genomics, mito-nuclear discordance, introgression, balancing selection

## Abstract

Cystic echinococcosis (CE), caused by *Echinococcus granulosus sensu stricto* (*s.s.*), poses a substantial risk to both humans and domestic animals globally. Here, we compared the whole genomes of 111 *E. granulosus s.s.* samples from China. Genomic variation data revealed frequent cross-fertilization in the hermaphroditic *E. granulosus*. The G1 and G3 genotypes represent distinct mitochondrial lineages, while showing no differentiation in the nuclear genome, suggesting mito-nuclear discordance caused by historical geographic separation and subsequent fusion. Population structure, demographic history, and gene flow among populations reflected the transmission route of *E. granulosus s.s.* from the Middle East to Qinghai-Xizang Plateau through the migration of nomadic people, followed by introgression during secondary contact. Genomic variations highlighted selection signatures within the genome prone to balancing selection, particularly impacting genes encoding membrane-related proteins, representing a potential evolutionary strategy for adaptation to parasitic life. Balancing selection pressure on the gene-coding sodium/bile acid cotransporter led to its high level of genetic stability, which may play a crucial role in the survival and development of *E. granulosus* during the parasitic stage, making it a potential drug target for the treatment of CE. Meanwhile, other genomic regions under strong balancing selection may provide potential targets for protective immunity. These findings offer valuable insights into the complex dynamics and adaptive evolution of *E. granulosus s.s.* in China.

**IMPORTANCE:**

*Echinococcus granulosus sensu stricto* (*s.s.*) is the primary cause of cystic echinococcosis (CE), a parasitic disease affecting humans and livestock with significant health and economic impacts. Previous studies on this parasite relied on mitochondrial DNA to classify its genotypes and understand its genetic diversity. However, these studies cannot capture the full complexity of its evolutionary dynamics and adaptation strategies. Our research employs comprehensive genome-wide sequencing, offering a more nuanced view of its genetic landscape. We discovered that cross-fertilization appears to be a prevalent reproductive strategy in the hermaphroditic *E. granulosus*, underpinning the observed deep mitochondrial divergence between genotypes G1 and G3, as well as gene flow among populations. The transmission history of *E. granulosus s.s.* in China and its widespread genetic mixing were likely facilitated by the migrations of nomadic peoples. Furthermore, we identified genes under balancing selection, including the gene involved in the uptake of host bile acids, which play a crucial role in the parasite’s survival and development, potentially offering new targets for intervention. Our research advances the understanding of the genetic diversity and evolutionary strategies of *E. granulosus*, laying the foundation for improved control measures of CE.

## INTRODUCTION

Cystic echinococcosis (CE) poses a substantial risk to both humans and domestic animals worldwide. As one of the most hazardous food-borne parasitic diseases (1), CE causes one million disability-adjusted life years, with an economic toll of $4.11 billion annually (2–4). This disease is caused by the ingestion of eggs from the *Echinococcus granulosus sensu lato* complex, which parasitizes the small intestine of dogs and other wild carnivores (5, 6). Previously, the species complex of *E. granulosus* was classified into genotypes G1–G10 and *Echinococcus felidis* based on morphology and mitochondrial (mt) genome sequences (7–11), with genotypes G2 and G9 later considered invalid (12, 13). Among them, *E. granulosus sensu stricto* (*s.s.*; genotypes G1 and G3) is responsible for approximately 88% of human CE cases worldwide (14).

Recent mt genetic studies have shed light on the genetic diversity and molecular epidemiology of *E. granulosus s.s.*, enhancing evolutionary understanding toward the control and surveillance of this parasite (15–21). Notably, the comparison of near-complete mitogenomes from samples worldwide has unveiled high genetic diversity and insights into the global transmission routes of *E. granulosus s.s.* (22).

Out-crossing results in mito-nuclear discordance (23). Therefore, given the complex reproductive biology of *E. granulosus*, involving asexual reproduction in larval stage and sexual reproduction in adults (24), mitochondrial sequences alone might not be sufficient to accurately infer evolutionary relationships among populations. For instance, despite fixed differences between the mt genomes of G1 and G3 (13, 22), the lack of genetic differences at three nuclear loci suggested phylogenetic discrepancy between mt and nuclear markers (13). This highlighted the need for a comprehensive whole-genomic comparison to unravel the intricate evolutionary dynamics of this parasite.

The recent genomic resources have greatly facilitated the comparative and functional studies of *E. granulosus*, shedding light into the parasitism adaptation, nutrient metabolism, and the identification of potential drug targets (25–28). However, the paucity of genome-wide variation data prevents further understanding of reproductive biology, dispersal history, and recent adaptation among *E. granulosus* populations.

In the present study, we performed a pioneering genome-wide survey of *E. granulosus s.s.*, focusing on populations from key endemic regions in China. Our findings revealed population structure, mito-nuclear discordance, gene flow, and selection signatures, offering insights into the parasite’s transmission routes, reproductive strategies, and adaptive genetic traits. The key genes under balancing selection provide potential targets for new control interventions. These results provided a deeper understanding of the complex evolutionary dynamics and adaptive mechanisms of this parasite.

## RESULTS

### Detection of genome-wide polymorphisms

**Fig 1:**
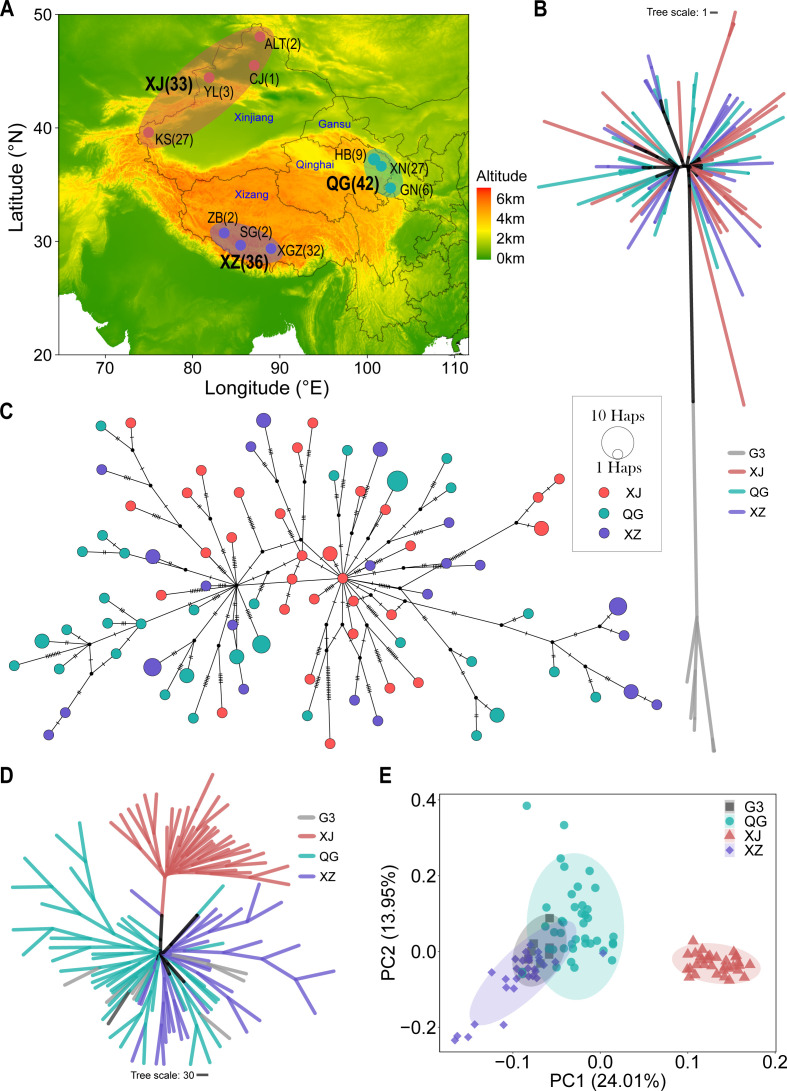
Geographic locations and phylogenetic reconstruction of *E. granulosus s.s.* populations. (A) Geographic distribution of the three *E. granulosus s.s.* populations. The numbers in parentheses indicate the quantity of samples. The uppercase letters represent abbreviations for population and location names. KS, Kashgar; YL, Yili; CJ, Changji; ALT, Aletai; HB, Haibei; XN, Xining; GN, Gannan; ZB, Zhongba; SG, Saga; XGZ, Xigaze. The text in blue font denotes the provinces involved in this study’s sampling. Elevation data and administrative boundaries of China were generated using the publicly available raster and maps packages in R. (B) The neighbor-joining (NJ) phylogenetic tree for mt genomes. (C) mt Haplotype network analysis of the G1 genotype by the median-joining network method. (D) The NJ phylogenetic tree for the thinning SNPs data set of nuclear genome. (E) Principal component analysis (PCA) for the thinning SNPs data set of nuclear genome.

Through quantitative PCR (qPCR) identification, 111 *E. granulosus s.s.* samples in China with less than 5% host genome contamination were selected for downstream analyses (Table S1). Based on geographic distribution, the samples were initially categorized into three groups: Xinjiang (XJ; *n* = 33), Qinghai and Gannan (QG; *n* = 42), and Xizang (XZ; *n* = 36; Fig. 1A and Table S1). Whole-genome sequencing (WGS) of all samples generated a minimum of 35.16-fold and an average of 298.55-fold sequencing depth (Table S1). Variant calling of the genomic data revealed 715,521 high-quality single nucleotide polymorphisms (SNPs) across the entire genome (Table 1). Not surprisingly, SNP density was higher in intergenic regions and lower in exonic regions (Table 1). This distribution pattern was linked to chromosome structure, where SNP density showed an inverse relationship with gene density but was positively correlated with repeat sequence density (Table S1). For instance, chromosome Chr3 (CM038614.1) exhibited the lowest polymorphism density due to its more gene-rich regions and fewer repeat-rich regions, while chromosome Chr6 (CM038617.1), contrary to Chr3, had the highest SNP density (Fig. S1 and Table S2). Group-specific SNPs were also identified, with the XJ group displaying the highest number of unique SNPs, despite its smaller sample size (Fig. S2).

**TABLE 1:** Summary of the SNPs identified in *E. granulosus s.s.* among all investigated samples and three different populations

Population	All	XJ	QG	XZ
Sample count	111	33	42	36
SNPs	715,521	664,734	671,420	642,160
Intergenic	564,165	532,644	535,956	517,769
Downstream	152,893	134,982	137,758	126,994
Upstream	158,223	139,535	142,022	130,309
Exon	27,979	23,730	24,953	22,808
Missense	15,016	12,442	13,274	12,190
Intron	121,242	106,516	108,615	99,847
Splicing	2,135	1,844	1,896	1,736

### Mito-nuclear discordance between genotypes G1 and G3

The phylogenetic tree of mt genomes clearly revealed distinct genetic differentiation between genotypes G1 and G3, but no geographic structuring was observed within genotype G1 (Fig. 1B; Fig. S3A). A mt haplotype network was generated for G1 samples, revealing a similar absence of clear geographic differentiation (Fig. 1C). However, the samples from Xinjiang appeared to represent more ancestral haplotypes, from which other regional haplotypes diverged (Fig. 1C). This suggested that Xinjiang might have served as an origin for the subsequent spread of genotype G1. Similarly, the phylogenetic tree with the globally published mt genomes also indicated a lack of geographic structuring within the genotype G1 (Fig. S4). In contrast, for nuclear genomes, the phylogenetic tree (Fig. 1D; Fig. S3B) and principal component analysis (PCA; Fig. 1E) showed that the nuclear genomes of genotype G3 clustered closely with geographically related samples with genotype G1 despite the distinct differentiation of the mt genome (Fig. 1B). The result verified the mito-nuclear discordance between G1 and G3 genotypes, indicating that they were merely different mt genotypes, rather than different nuclear genotypes, which represent a typical case of deep mitochondrial divergence (DMD) (23, 29). This can be attributed to the “divergence reversal” phenomenon, where they underwent an ancient period of isolation and divergence, followed by recent frequent nuclear gene flow, leading to genomic homogenization. However, the non-recombining mt genome retained its original genetic variation (29).

### Population structure

To assess how these three geographical groups should be genetically classified, we applied various population structure analyses to evaluate the genetic differentiation both between and within these groups. The phylogeny based on nuclear genomes revealed a clear geographic structure: the XJ group formed a distinct monophyletic clade, while the QG and XZ groups, although not forming distinct monophyletic branches, showed geographic clustering (Fig. 1D; Fig. S3B). Similarly, the PCA of nuclear SNPs clearly separated the XJ group from the XZ and QG groups along PC1, while the 95% CIs of XZ and QG groups exhibited partial overlap (Fig. 1E). Further PCAs within each group indicated that, despite the presence of a few outliers, no geographic structure within groups was detected (Fig. S5), which was also confirmed by nuclear genome phylogeny (Fig. S3B). The low proportion of variance explained by PC1 and PC2 (Fig. S5) also suggested a lack of genetic differentiation within the three groups.

**Fig 2:**
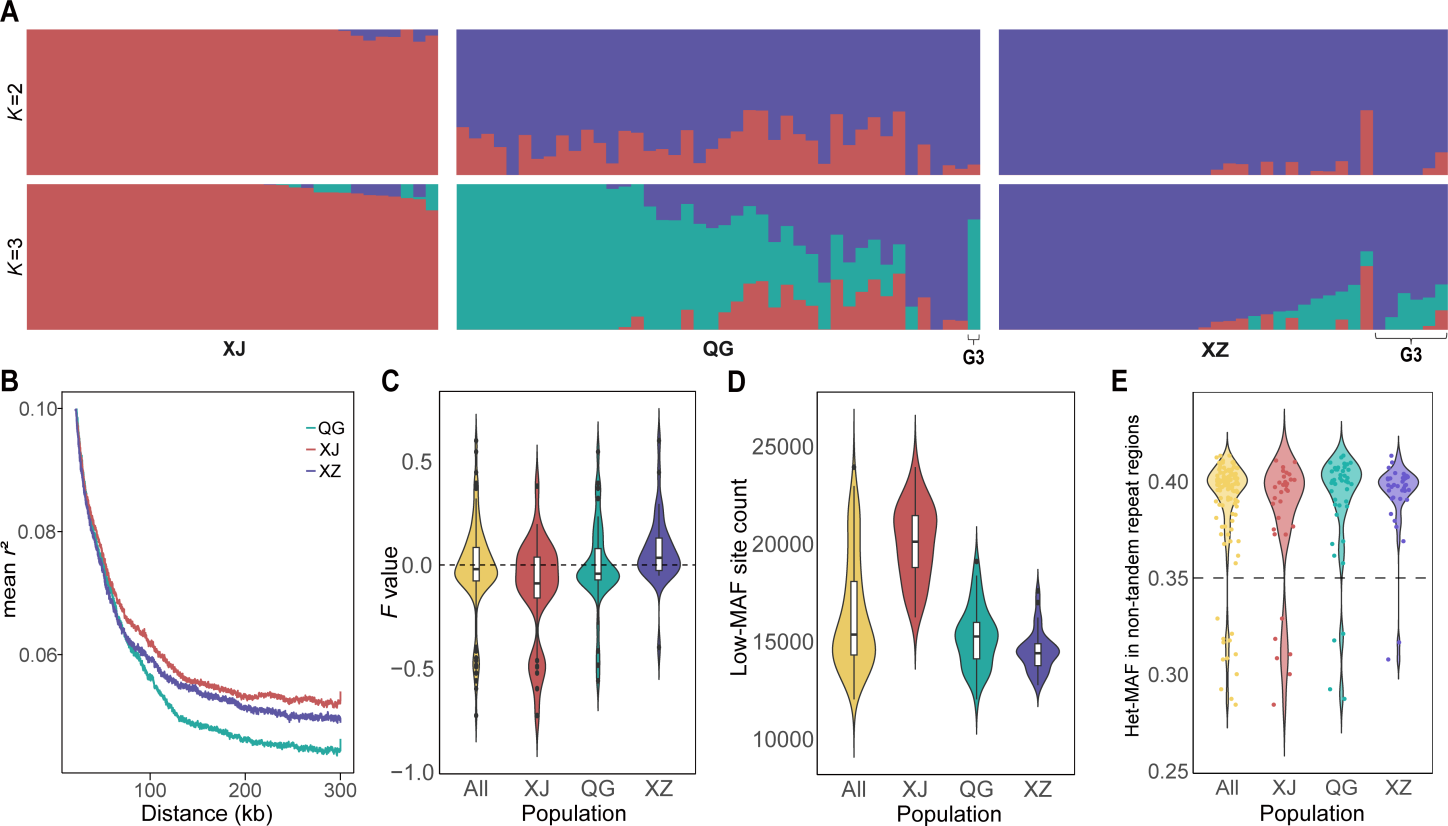
Population structure of *E. granulosus s.s.* populations. (A) Admixture analysis showing the genomic ancestry composition of the three populations with the *K* = 2 and 3. (B) Linkage disequilibrium (LD) decay in different populations of *E. granulosus s.s.* (C) Violin plot of *F* values (inbreeding coefficients) for all samples and the three populations. (D) Violin plot of low minor allele frequency (Low-MAF) site counts for all samples and the three populations. (E) Violin plot of overall minor allele frequency at heterozygous sites (Het-MAF) within non-tandem repeat regions for all samples and three populations.

The ADMIXTURE analysis unveiled the genomic ancestry composition underlying population differentiation across geography. By testing *K* ranging from 1 to 10, the lowest cross-validation (CV) error pointed to two distinct clusters (*K* = 2) of genomic composition (Fig. 2A and Fig. S6). Under this scenario, the XJ group exhibited minimal genetic sharing with the other two groups (Fig. 2A), suggesting that it should be considered as a genetically distinct population. Meanwhile, the QG group displayed a higher proportion of genetic components from the XJ population, and the XZ group also showed few genetic influences from the XJ population (Fig. 2A). It appears that the genetic differentiation between QG and XZ was likely due to varying degrees of genetic admixture involving the XJ population or incomplete lineage sorting (ILS). However, it is noteworthy that under the *K* = 3 scenario, a relatively low CV error was also observed (Fig. S6), and the major genetic components of QG and XZ were differentiated (Fig. 2A). This indicated that the inherent genetic components of QG and XZ groups have also begun to diverge. This divergence appears to be recent, as they have not yet developed into fully genetically independent populations (Fig. 1D and E). For *K* > 3, the CV error increased rapidly (Fig. S6), and no further meaningful or structured subdivision of genetic components was observed across populations and genotypes (Fig. S7). In summary, we classified the QG and XZ groups as distinct populations to more accurately reconstruct the detailed evolutionary history of geographically isolated populations.

Genetic recombination disrupts the linkage between alleles, causing a decay in linkage disequilibrium (LD) along genomic positions (30). In this study, the overall LD decay trends were similar across the three populations (Fig. S8). However, a magnified view revealed that LD decay rates are closely related to recombination rates and genetic admixture within the populations (Fig. 2A and B; Fig. S9B; Table 2). The QG population may have experienced greater genetic admixture from XJ and XZ populations (Fig. 2A), which could have contributed to this elevated recombination rate (Table 2) and faster LD decay (Fig. 2B). In contrast, the XJ population, with a lower recombination rate (Table 2), showed slower LD decay (Fig. 2B), likely due to the limited genetic admixture from other populations (Fig. 2A).

**TABLE 2:** The mean genomic pairwise Wright’s *F* statistics (*F*_ST_) between populations, and recombination rate (*r*), nucleotide diversity (π), and effective population size (*N_e_*) within populations, as well as the 95% CIs for each index

Population	*F*_ST_ (10^−2^)	*R* (cM/Mb)	π (10^−3^)	*N_e_* (10^2^)
XJ	To XZ: 5.563 (5.506–5.618)	0.7501 (0.7243–0.7764)	1.058 (1.048–1.068)	114.5 (113.4–115,6)
QG	To XJ: 4.159 (4.112–4.206)	0.9269 (0.8936–0.9606)	0.931 (0.921–0.941)	100.8 (99.7–101.8)
XZ	To QG: 2.514 (2.485–2.544)	0.8059 (0.7760–0.8359)	0.929 (0.920–0.940)	100.5 (99.6–101.7)

Pairwise Wright’s *F* statistics (*F*_ST_, relative divergence index) of genetic variations further revealed that the largest genetic differentiation occurred between the XJ and XZ populations, followed by the differentiation between the XJ and QG populations, and, lastly, the differentiation between the XZ and QG populations (Table 2; Fig. S10A). Genetic diversity among the three populations was compared using nucleotide diversity (π) and effective population size (*N_e_*), which are directly proportional (31). The XJ population exhibited the highest π and the largest *N_e_*, while the XZ population showed the lowest levels of both (Table 2; Fig. S10B). *Schistosoma mansoni*, a dioecious species, has higher genetic diversity (~0.003) than hermaphroditic *E. granulosus* (~0.001), likely driven by its cross-fertilization reproductive strategy over the long process of evolution (32).

### Cross-fertilization reproduction in hermaphroditic *E. granulosus* s.s

The extent of cross-fertilization among hermaphroditic organisms determines the landscape of genome-wide LD decay, recombination, and heterozygosity. In this study, the distance of *r*^2^ for all three populations was reduced by half of its maximum value at around 4 kb (Fig. S8), which is significantly faster than the decay observed in dioecious *Schistosoma japonicum* from the Philippines (85 kb) and Taiwan (98 kb) populations (33). Additionally, the mean recombination rate of the *E. granulosus s.s.* genome is comparable to that of other cross-fertilization species, such as mouse (0.5–1.0 cM/Mb; Table 2; Fig. S9B) (34). In addition, low-gene-density regions on the chromosomes were more prone to recombination compared to high-gene-density regions, potentially introducing more heterozygous sites (Fig. S9). This observation aligns with patterns seen in some cross-fertilization species (35). We also noted a notable level of unexpected heterozygosity in certain samples (*F* value < 0; Fig. 2C). These results suggested that hermaphroditic *E. granulosus* has the capacity for cross-fertilization reproduction. In most samples, the proximity to expected heterozygosity under Hardy-Weinberg equilibrium (*F* value ≈ 0; Fig. 2C) indicated that their mating preference is similar to that of the random mating organisms (36). Nonetheless, the high inbreeding coefficients (*F* value > 0; Fig. 2C) were observed in some samples, suggesting that mating between close relatives or self-fertilization also occurs (36), which could be attributed to the low within-host worm density or local parasitic dispersal.

### Factors affecting the Low-MAF site in samples

Contamination between samples or polyploidy may lead to an increase in the number of low minor allele frequency (Low-MAF) sites (37, 38). In this study, we observed a distinct population structure in the counts of Low-MAF sites among samples from different populations (Fig. 2D). Specifically, samples from the XJ population generally contained a higher number of Low-MAF sites, while those from the XZ population exhibited the lowest counts (Fig. 2D). For each pure diploid individual, the total minor allele frequency of heterozygous sites (Het-MAF; i.e., the ratio of the reads of minor alleles to the total reads at heterozygous sites) in non-tandem repeat regions is expected to be close to but below 50%. In contrast, this frequency is significantly lower in polyploid or mixed individuals. In our analysis, 12 samples exhibited values around 30%, which were noticeably lower than the approximately 40% observed in other samples (Fig. 2E). Further examination of the density distribution of alternate allele frequencies for each SNP revealed that four of them were polyploid (Fig. S11), a phenomenon also reported in the genome of *Echinococcus multilocularis* (26). The other eight samples showed admixture of distinct genetic backgrounds (Fig. S12). However, these polyploid and mixed samples were not the primary drivers of the population structure observed in Low-MAF site counts (Fig. 2D and E). Meanwhile, the Het-MAF in non-tandem repeat regions of samples showed a relatively low correlation with their corresponding Low-MAF site counts (*R* = 3.1; Fig. S13A). Importantly, these mixed samples did not deviate from their respective populations in terms of phylogenetic relationships or population structure (Fig. 2A; Fig. S3B and S14). This suggested that the mixing occurred within the population, reflecting the natural genetic variation and inherent genetic diversity of the population. Therefore, these samples were retained in all analyses conducted in this study.

Further analysis revealed a significant positive correlation between Low-MAF site count and both π and heterozygous site count in each sample and a significant negative correlation between Low-MAF site count and *F* values of inbreeding coefficient (Fig. S13B through D). This indicated that the larger effective population size and higher nucleotide diversity in the XJ population (Table 2) contributed to the increase of heterozygosity and Low-MAF site (Fig. 2C and D; Fig. S13B through D). In contrast, the smaller effective population size and lower nucleotide diversity in the XZ population (Table 2) made it more susceptible to genetic drift (39), resulting in reduced heterozygosity and fewer Low-MAF sites. Therefore, the primary factors driving the differences in Low-MAF site count between populations are variations in effective population size and genetic diversity.

### Demographic history of *E. granulosus s.s.* populations

**Fig 3:**
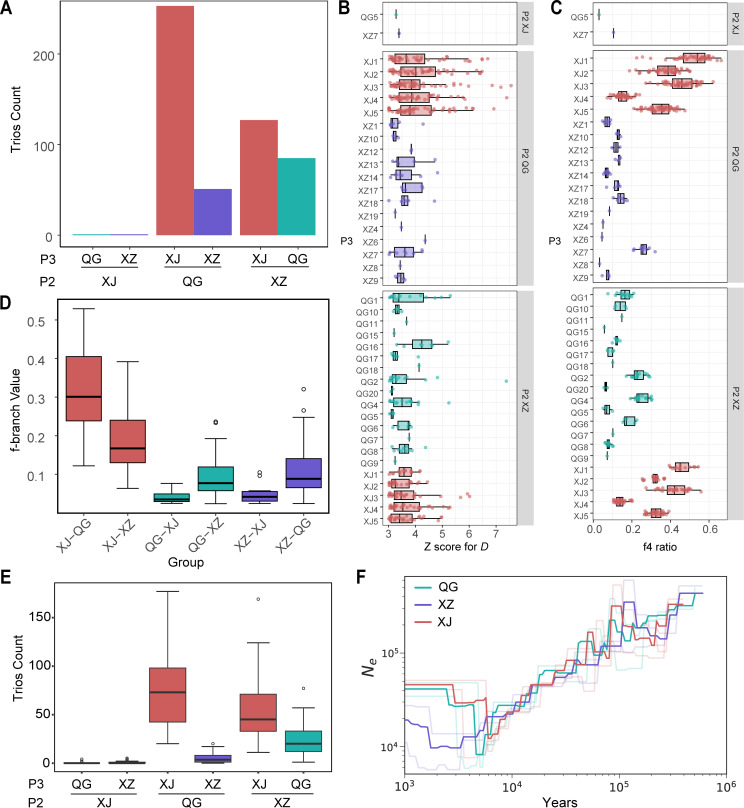
Demographic history of *E. granulosus s.s.* populations. (A) The histogram illustrates the number of trios between different populations in the *D*-statistics, representing the reliable gene flow (BH-corrected *P*-value < 0.05 and *Z* score for *D* > 3) from P3 to P2 based on population structure grouping in Table S3. (B) Standardized *Z* scores of *D* for all trios in Fig. 3A, illustrating gene flow from different P3 subgroups in Table S3 to their corresponding P2 populations. (C) The f4-ratio for all trios in panel A illustrates the strength of gene flow from different P3 subgroups in Table S3 to their corresponding P2 populations. (D) The *f*-branch (*f_b_*) metric identifies the extent of excess allele sharing between populations, as related to Fig. S14. (E) The box plot depicts the count of trios contributing to gene flow from P3 to P2 in the *D*-statistics based on 50 independent runs of the random groupings. (F) Inferred effective population size (*N*_e_) over time for the three populations using subcommand CV in SMC++ with default 100 bp window size. The shaded lines were generated by twofold CV, while the solid lines indicate their best fit.

Genetic admixture between populations may arise from ILS or gene flow. Therefore, we employed multiple tests to ascertain the contribution of gene flow to genetic admixture between populations (Fig. 2A). According to the grouping criteria of population structure (Table S3), the *D*-statistics (ABBA-BABA test), known for its robustness in detecting genome-wide gene flow bias (40–42), identified 699 reliable gene flow trios with positive *D* representing P3 to P2 (Benjamini–Hochberg [BH]-corrected *P*-value < 0.05 and *Z* score for *D* > 3; Fig. 3A). The trios representing gene flow bias from XJ to QG were the most frequent and exhibited stronger robustness (Fig. 3B; Fig. S15A). Significantly more trios were also detected with the gene flow bias from XJ to XZ, as well as between QG and XZ, whereas from QG and XZ to XJ was minimal (Fig. 3A). The relative intensity of gene flow, assessed using f4-ratio and *f*-branch (*f_b_*), also showed that the gene flow from XJ to QG was strongest, followed by the gene flow from XJ to XZ and between QG and XZ, while the gene flow from QG and XZ into XJ was relatively weak (Fig. 3C and D; Fig. S15B and S16). Furthermore, the statistical analysis of *D*-statistics results from 50 random groupings (Fig. 3E) consistently revealed the distribution scenario of gene flow contributions above, validating the robustness of gene flow detection based on population structure grouping (Table S3). The analysis results here corroborated that gene flow between populations is the main factor leading to genetic admixture among populations (Fig. 2A).

Historical changes in effective population size (*N*_e_) calculated by SMC++ revealed similar trends of demographic history across the three populations, with *N_e_* gradually decreasing around 100 thousand years ago (kya), followed by an expansion after the bottleneck period in the recent few thousand years (Fig. 3F). Among the three populations, the XJ population first experienced the bottleneck event (around 5–8 kya), followed by the QG population (around 4–6 kya), and finally the XZ population (around 1–2 kya; Fig. 3F). These findings were consistent across different window sizes calculated by SMC++ (Fig. S17). Considering the founder effect of population colonization in previously unoccupied areas, the sequential population bottleneck may reflect the order of population establishment during the dispersal of *E. granulosus* s.s (43, 44). This reflected a phenomenon where the earlier a population colonized, the longer it developed (Fig. 3F), leading to greater nucleotide diversity and a larger effective population size (Table 2).

### Absence of environmental adaptive evolution across populations

Due to the unclear background of naturally infected hosts and the absence of observable phenotypic differences, we exclusively explored the genomic characteristics of adaptation to different environments. Utilizing across-population approaches (*F*_ST_ and XP-*n*S_L_), we compared the XJ population inhabiting a temperate continental climate with the XZ and QG populations residing in a highland monsoon climate. The *F*_ST_ analysis revealed that the same number of genes (*n* = 340) overlapping significant differentiation windows between QG and XJ populations, as well as between the XZ and XJ populations (Tables S4 and S5; Fig. S10). The XP-*n*S_L_ analysis identified 163 genes in the XZ population and 227 genes in the QG population that overlapped with the candidate selection windows (Tables S4 and S5; Fig. S18). The overlapping genes detected by both methods in the QG and XZ populations were significantly enriched in the category related to the plasma membrane (GO:0005886; Tables S6 and S7). However, there was an absence of linked selection sites related to these enriched genes. We further investigated the environmental stress-activated ECG_03343 (coding mitogen-activated protein kinase 14, MAPK14) and ECG_03345 (coding mitogen-activated protein kinase 11, MAPK11) genes (45), which were involved in multiple pathways (Table S8). Similarly, no linked selection sites were identified in these two genes.

The absolute divergence index (*D*_xy_) did not exhibit a higher difference in gene-enriched regions compared to repeat sequence regions (Fig. S1), as observed with *F*_ST_ (Fig. S10 and S18). This result suggested that the divergence between populations may be primarily associated with the recent reduction of gene flow or haplotype distribution of ancestral divergence (46, 47), rather than directional selection under different environmental pressures.

### Genomic balancing selection indicated adaptation to the host internal environment

In view of the above, coupled with the similar distribution patterns of genomic π and selection signals across populations (Fig. S10 and S18), we treated the three populations as a cohesive population and employed within-population approaches (Tajima’s *D* and *n*S_L_) to explore the evolutionary trends of *E. granulosus s.s.*

**Fig 4:**
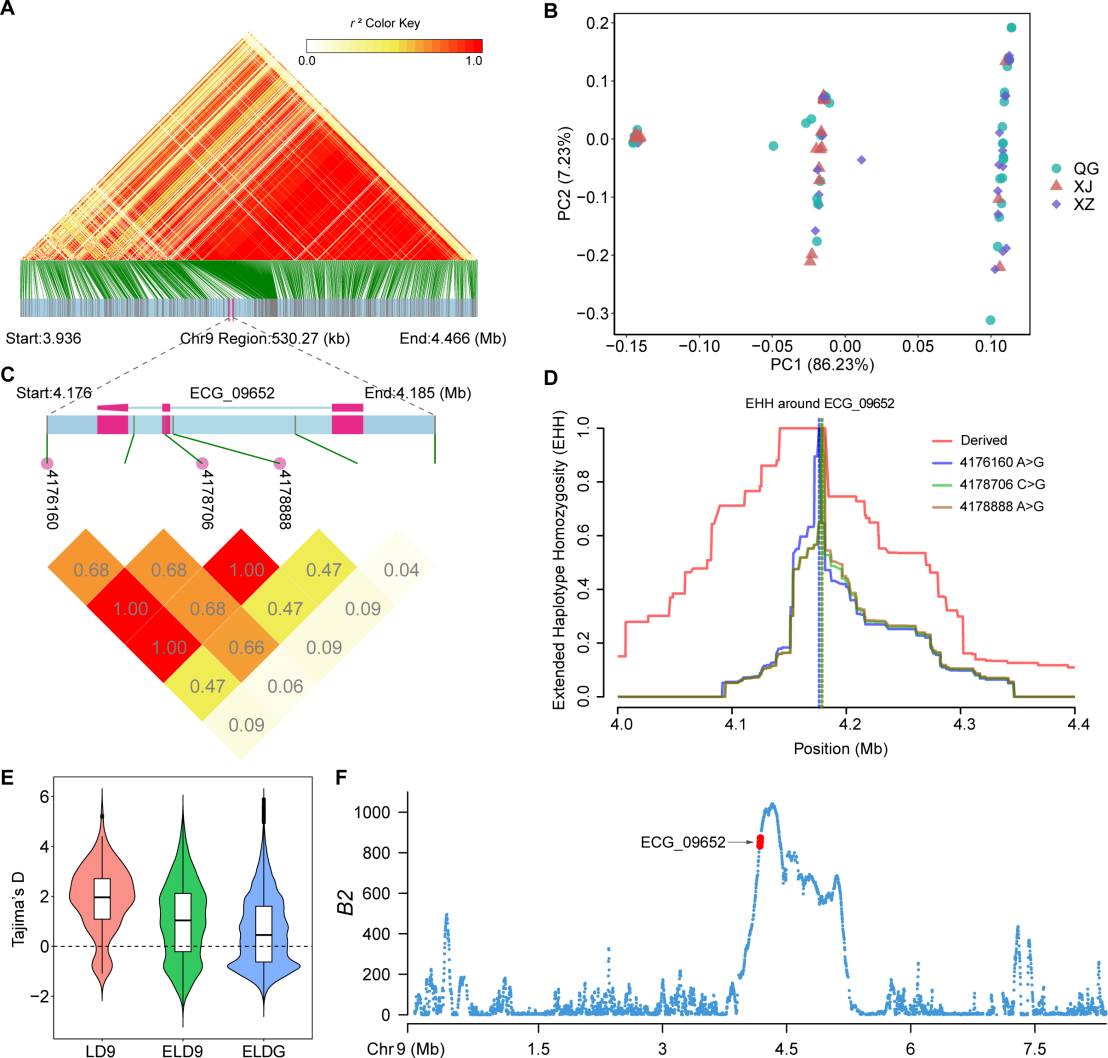
The ECG_09652 gene and its surrounding regions are subjected to strong linked selection. (A) LD heatmap of the region with 530.27 kb length around the ECG_09652 gene on Chr9. (B) PCA of the LD region is shown in panel A. (C) LD heatmap of the ECG_09652 gene and its upstream and downstream regions. The SNP sites of Chr9- ECG_09652–4176160, 4178706, and 4178888 were under strongly linked selection (*r*^2^ = 1.00). (D) Extended haplotype homozygosity (EHH) decay around the three SNP sites of Chr9- ECG_09652–4176160, 4178706, and 4178888. The three sites exhibit overlapping derived red curves. (E) Violin plot of the distribution of Tajima’s *D* values for 2 kb non-overlapping windows in the LD region shown in panel A and the other regions of the genome. Filter out all windows without SNP before plotting. LD9, the LD region shown in panel A on Chr9. ELD9 excludes the LD region shown in panel A on Chr9. ELDG excludes the LD region shown in panel A on the whole genome. The dashed lines represent a horizontal line at zero value. (F) Scatter plot illustrating *B*_2_ statistics across Chr9 for the entire population, calculated along non-overlapping windows of 2 kb. The points highlighted in red correspond to the windows overlapping with the ECG_09652 gene.

In both Tajima’s *D* and *n*S_L_ analyses, the 674 genes that overlapped with the top 1% windows (Fig. S19 and Table S9) did not exhibit significant enrichment in any Kyoto Encyclopedia of Genes and Genomes (KEGG) pathway (Table S10). In the gene ontology (GO) enrichment analysis, many genes showed significant enrichment in GO terms related to the membrane (GO:0016020; Table S11), indicating their adaptation to the parasitic lifestyle. Among those genes, the ECG_09652 gene (encoding sodium/bile acid cotransporter, SBAT) participates in the uptake of bile acids from the host’s intestines, which is essential for the development of *E. granulosus* (25). Three sites under strongly linked selection were identified in the putative promoter region (Chr9-4176160, A > G), exon (Chr9-4178706, C > G), and intron (Chr9-4178888, A > G) of the ECG_09652 gene (Fig. 4C and D). The mutation at Chr9-4178706 in the exon (Fig. 4C) was a synonymous mutation. These three sites divided all samples into three haplotypes: ACA/ACA homozygotes, GGG/GGG homozygotes, and ACA/GGG heterozygotes. The ECG_09652 gene was situated within a contiguous LD region on Chr9 spanning a length of 530.27 kb (from Chr9-3935911 to Chr9-4466186; Fig. 4A). Within this LD region, the median value of Tajima’s *D* was higher compared to other regions on Chr9 and the entire genome (Fig. 4E). Additionally, calculations based on *B*_2_ statistics (Fig. 4F) confirmed that the LD region on Chr9 was under strong balancing selection. The PCA of SNPs within this region revealed that due to the LD between sites, PC1 can also partition all samples into three haplotype populations highly consistent with the three haplotypes of the ECG_09652 gene but unrelated to geographical distribution (Fig. 4B). This indicated that the balancing selection of the ECG_09652 gene and its linked sites is not caused by different geographical environments or geographic isolation but rather may stem from adaptation to the host internal environment. The SBAT, encoded by ECG_09652, facilitates the entry of bile acids into cells, which can stimulate the parasite’s development (25), but excessive concentrations of bile acids are toxic to the parasite (48, 49). Therefore, it is hypothesized that under balancing selection pressure, mutations in ECG_09652 do not affect its function, maintaining high genetic stability to ensure bile acid homeostasis within the parasite.

**Fig 5:**
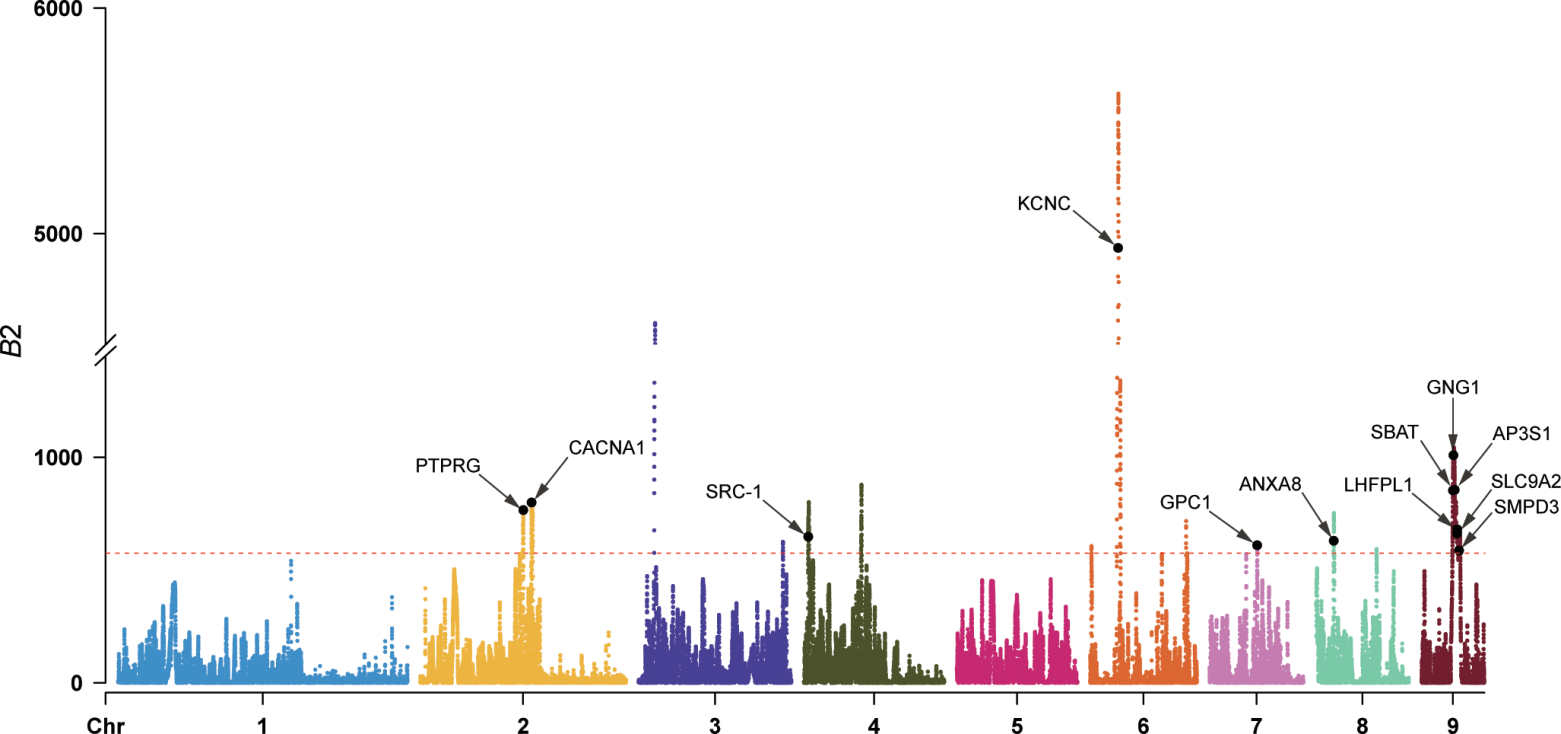
Balancing selection signals based on *B*_2_ statistics calculated along non-overlapping 2 kb windows for all samples in this study. The red dashed line represents the threshold of the top 1% value. The black dots represent the representative windows for surface protein-coding genes under balancing selection, related to Table S12.

Finally, we identified a total of 11 additional surface protein-coding genes under balancing selection by overlapping with the top 1% of windows based on *B*_2_ statistics (Fig. 5 and Table S12). Balancing selection pressure on parasite surface proteins typically arises under host immune pressure (50), making them potential targets for immune protection.

## DISCUSSION

A crucial aspect of controlling parasitic diseases is comprehending the historical patterns and driving factors influencing pathogen transmission and adaptation. Despite the widespread use of mt markers as the primary data source for phylogenetic studies of *E. granulosus*, our study’s genome-wide variation data offer reliable and unbiased information to address issues related to CE control.

### Cross-fertilization in *E. granulosus*

The life cycle of *Echinococcus* spp. involves asexual reproduction of larvae and sexual reproduction of adults (8). Morphological studies provided evidence that adult worms predominantly utilize cross-fertilization for reproduction, resorting to self-fertilization only when cross-fertilization is impractical (51, 52). This study offers multiple lines of genomic evidence supporting cross-fertilization in *E. granulosus*. Examples include the mito-nuclear discordance between G1 and G3 genotypes (Fig. 1B through E; Fig. S3), the rapid LD decay of genome (Fig. 2B), the high recombination rate and recombination structure distribution of genome (Table 2; Fig. S9), unexpected heterozygosity in some samples (Fig. 2C), and the gene flow between populations (Fig. 3A through E; Fig. S15 and S16). Recent studies also unequivocally established that multiple infections often result in the co-existence of adults with distinct genetic backgrounds or even different *Echinococcus* species within definitive hosts (24, 53–56). Individuals exhibiting elevated inbreeding coefficients (*F* value > 0; Fig. 2C) further indicate the presence of inbreeding or self-fertilization, resulting from the conspecific fertilization among adults with identical genetic backgrounds derived from asexual reproduction (24).

### The mito-nuclear discordance

Recent studies have highlighted instances of discordance between mitochondrial DNA (mtDNA) and nuclear DNA (nuDNA) (23). The DMD pattern of mito-nuclear discordance could be attributed to several hypotheses. In long-lived self-fertilization individuals, DMD can occur due to the faster mutation rate of mtDNA compared to nuDNA (23, 57). However, this explanation seems unlikely for the DMD here, given the absence of corresponding shallow genetic differentiation in the nuclear genome (Fig. 1D and E; Fig. S3B). Another scenario involves the secondary contact of previously geographically isolated lineages. Before the frequent nomadic activities of humans, the G3 genotype likely diverged from the G1 genotype due to geographic isolation, eventually becoming the dominant population in the South Asian subcontinent (20, 58). Subsequently, frequent gene flow caused by historical human nomadic and trade activities led to the homogenization of their nuclear genomes. Simultaneously, parthenogenetic inheritance of mitochondria could have retained the original genetic variation (23). The third scenario, termed “ghost introgression,” suggests that the mt genome of G3 may represent remnants of an extinct genotype and captured through genetic introgression (23, 29), analogous to the introgression from Neanderthals (*Homo sapiens neanderthalensis*) and Denisovans (*Homo sapiens denisova*) into the modern human genome (59, 60). However, it is challenging to rule out the existence of individuals with ancestral G3 nuclear genomes or those in an early stage of “divergence reversal,” particularly in the South Asian subcontinent where the G3 genotype predominates (20, 58). Further validation is required to confirm the existence of G3-specific nuclear genomes in these last two biogeographic scenarios.

In contrast to the DMD observed between genotypes G1 and G3, the mt genomes displayed weaker geographic structuring (Fig. 1B and C; Fig. S3A). Indeed, the mt genomes of G1 from various regions showed no geographic structuring, except for samples from South America (Fig. S4) (22). The mito-nuclear discordance observed across different geographic populations can be attributed to the greater number of phylogenetically informative sites in the whole nuclear genome compared to the mt genome. This discrepancy allows for the detection of potentially subtle differentiations (23).

### Unraveling the transmission history of *E. granulosus s.s.* in China

The transmission of CE is closely linked to animal husbandry, given that *E. granulosus* primarily reproduces through a complex life cycle involving domestic dogs and livestock (6). The early spread of *E. granulosus* has been proposed to be associated with the migration of livestock in the Middle East during the Neolithic Age (22). In this study, the demographic history also suggested that the initial population of *E. granulosus s.s.* in China was introduced along with the migration of sheep populations from the Middle East. Based on archaeological and genetic studies of sheep populations, Chinese domestic sheep are believed to have been introduced from the Middle East around 5–7 kya through the migration of pastoralist communities (61), with Xinjiang being the first destination along this route. Notably, the XJ population of *E. granulosus s.s.* was also the first population to undergo a founder effect around 5–8 kya (Fig. 3F), and the G1 mt genomes of the XJ population appear to be primitive haplotype (Fig. 1C).

**Fig 6:**
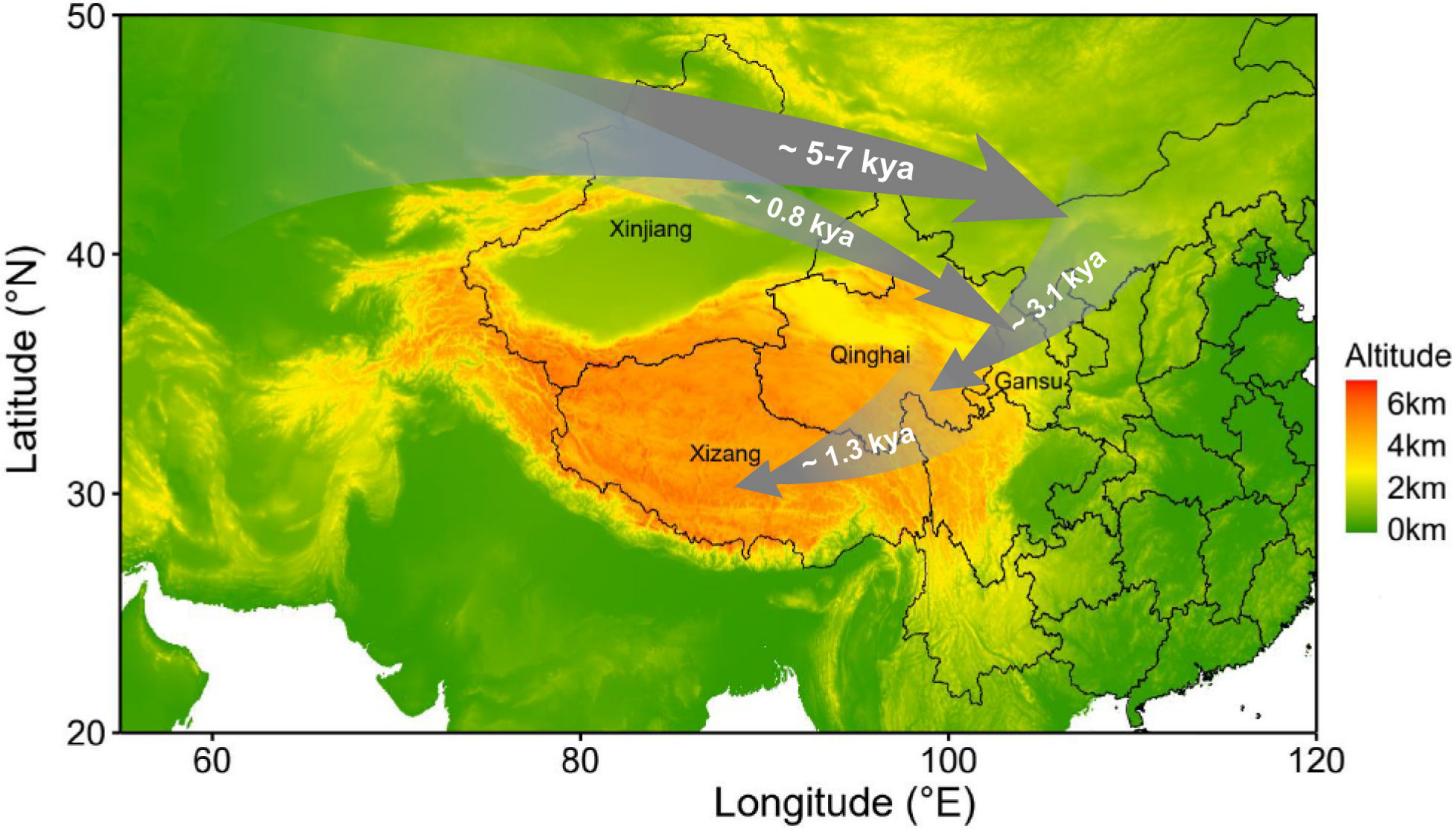
Inferred reconstruction diagram of the demographic history of *E. granulosus s.s.* in China. Elevation data and administrative boundaries of China were generated using the publicly available raster and maps packages in R.

The order of founder effects and the population structure relationships among these three populations (Fig. 1D and E and 3F; Table 2; Fig. S3B) indicated that the QG population acted as an intermediate step in the spread of *E. granulosus s.s.* from XJ to XZ. Therefore, the genetic data in this study support the transmission route of XJ → QG → XZ. The population genetic analysis of Tibetan sheep, combined with archaeological records of humans, livestock (such as sheep), and crops (such as wheat) in the Qinghai-Xizang Plateau (QZP) and surrounding areas, unveiled a two-step colonization pattern for the Tibetan sheep populations: around 3.1 kya from northern China to the northeast of QZP, followed by expansion from the northeast to the southwest of QZP around 1.3 kya (62). In this study, the timing of founder effects observed in the QG and XZ populations (Fig. 3F) coincide with the two-step colonization history of Tibetan sheep on the QZP (62). The genomic admixture (Fig. 2A) and gene flow from XJ into QG (Fig. 3A through E; Fig. S15 and S16) further indicated a secondary migration of the XJ population to the northeastern QZP. During the active Eurasian trade route (the Silk Road), starting around 2 kya, and the massive Mongol invasion of the western region 800 years ago, many Islamic peoples from the Arabian Peninsula, Persia, and Central Asia (including today’s Xinjiang) crossed the Silk Road to reach northern China actively or passively (61). These historical events may once again facilitate the spread of *E. granulosus s.s.* along the Silk Road to QZP. Additionally, the frequent interactions throughout history on the QZP—such as ethnic exchanges between groups like the Di-Qiang and Tibetans, nomadic movements as pastoralists migrated seasonally in search of grazing lands, and trade along routes such as the Tea Horse Road—further promoted gene flow between the QG and XZ populations (61, 63, 64), transmitting the genetic influence of the XJ population to the XZ population (Fig. 3A through E; Fig. S15 and S16). In light of the above, the transmission history of *E. granulosus s.s.* in China (Fig. 6) highlights the significance of *E. granulosus* as a noteworthy species, offering further insights into the history of sheep and human activities.

### The genetic differentiation among populations

We attempted to explore the adaptive evolutionary responses of the populations on the QZP to the plateau environment. Although genes responsible for population differentiation were enriched in the GO term related to the plasma membrane (GO:0005886; Tables S6 and S7), no distinct linked selection regions were identified at the chromosomal positions of these genes. Moreover, selective sweeps showed a lack of significant differences in the impact on the genome across the three populations (Fig. S19). This could be attributed to the relatively brief colonization history of the QZP populations, along with the stable living conditions within the host during the parasitic life stage. As a result, the *D*_xy_ values did not exhibit a higher difference in gene-enriched regions compared to the repeat regions (Fig. S18). Aligning with this perspective, the overall chromosomal profile of *E. granulosus s.s.* leaned toward a pattern of balancing selection (Fig. S19A and S20A), which sharply contrasts with the scenario observed in the free-living nematode *Caenorhabditis briggsae* (65). Based on these insights, we can infer that the present genetic differentiation among populations primarily stemmed from geographical isolation and recent reduction of gene flow, rather than adaptive evolution under environmental stress.

### Adaptive evolution of *E. granulosus* to parasitic lifestyle

Parasitic organisms rely heavily on membrane-related proteins for crucial functions, including host interactions, immune evasion, invasion, nutrient acquisition, and metabolite excretion (49). In this study, many genes under selection within the integrated population were significantly enriched in membrane-related GO term (GO:0016020; Table S11), indicating the potential evolutionary adaptability of *E. granulosus s.s.* to better thrive in its parasitic lifestyle. It is noteworthy that among these genes, the ECG_09652 gene maintained high genetic stability under balancing selection pressure, encoding the SBAT, which functions in the uptake of host bile acids. The larvae in the liver and the adult worms in the intestine are both exposed to high concentrations of bile acid, which can stimulate the parasite’s metabolism (66). Meanwhile, superfluous bile acids within the parasite are toxic and impact its survival (49). For instance, SBAT in *Clonorchis sinensis* maintains bile acid homeostasis in its adults, facilitating its survival in bile ducts (48). This underscores the need for *E. granulosus* to finely regulate the absorption and transport of bile acids to promote its development and adaptation to the parasitic environment. The expression levels of SBAT across different developmental stages of *E. granulosus* did not show significant differences (25, 26). These suggested that over the course of long-term evolution, *E. granulosus* retains stable SBAT expression across different developmental stages to ensure precise regulation of bile acid uptake. Inhibiting SBAT in *C. sinensis* can disrupt the bile acid homeostasis within its body, shortening the survival of its adults in bile (48). Thus, the SBAT of *E. granulosus* may represent a potential drug target for treating CE. Balancing selection within parasitic species is often driven by the host immune pressure, occurring more frequently on surface protein-coding genes (50). Therefore, the other surface protein-coding genes under balancing selection (Fig. 5 and Table S12) may be potential targets for protective immunity.

### Conclusion

In summary, we conducted a pioneering WGS analysis of *E. granulosus s.s.* populations, elucidating the intricate genetic dynamics between the genotypes G1 and G3, as well as among three different populations in China. These insights underscore the role of historical migration and geographic isolation in shaping the genetic landscape of *E. granulosus s.s.* We also noted that the genomes of different geographic populations lack signs of environmental adaptive evolution but exhibit distinct indications of balancing selection, which may indicate adaptation to the parasitic lifestyle. Genes under balancing selection offer potential drug or vaccine targets.

## MATERIALS AND METHODS

### Sample collection and DNA extraction

Livers and lungs with CE from yaks and sheep were collected during routine meat inspection at slaughterhouses in Xinjiang, Xizang, Qinghai, and Gansu provinces. The cyst surfaces were cleaned with normal saline, and protoscolices alone with cyst fluid from each cyst were extracted using a syringe and transferred to 15 mL or 50 mL tubes. After three rounds of precipitation and washing with phosphate buffer solution, the protoscolices in the tubes were transferred into freezing tubes and stored at −80°C in the laboratory for subsequent DNA extraction.

Subsequently, 50 µL of protoscolices from a single cyst of each host was used for DNA extraction using the Qiagen Blood and Tissue DNA Extraction Kit (Qiagen, Hilden, Germany), following the manufacturer’s instructions. All extracted DNA samples were then subjected to PCR amplification and sequencing as described by Ohiolei et al. (16) to distinguish the genotypes of the samples. After genotyping, all DNA samples of *E. granulosus s.s.* were further analyzed for the proportion of host genome contamination by qPCR using primers targeting single-copy genes in the host and parasite genomes (Table S13). Samples with parasite genomes accounting for more than 95% were selected for 100 bp paired-end sequencing on the BGISEQ500 (PE100) platform.

### Alignment, variant calling, and annotation

Sequencing reads were ﬁltered by removing adaptors and low-quality bases using fastp v0.19.3 (67). Only qualiﬁed pair-end reads were mapped to the reference genome of *E. granulosus s.s.* (GenBank assembly GCA_021556725.1 of genotype G1) (28) using BWA v0.7.17 with MEM algorithm (68) with default parameters. The resulting binary alignment/map (BAM) files were normalized to a consistent sequencing depth across samples by downsampling high-depth reads using the subsample parameter in samtools v1.16 (69), ensuring that high-depth samples were normalized to the overall mean depth, while lower-depth samples were unaffected. Variants were detected using FreeBayes v1.3.6 (70) with GCA_021556725.1 (28) as the nuclear reference genome and GenBank no. NC_044548.1 (71) as the mt reference genome. Using VCFtools v0.1.15 (72), we excluded sites with missing rates >20%, non-biallelic sites, and minor allele counts greater than or equal to 3 in all samples. To improve SNP accuracy, we also excluded sites where the mean depth was either lower than 5 or higher than three times the overall mean depth. Functional annotation of SNPs was performed using SnpEff v4.3 (73) with a self-constructed database using genome and annotation files of the reference genome.

### Genetic structure analyses

To perform population structure analysis, we generated a thinned SNP subset by selecting one variant within each 5 kb interval to eliminate LD using VCFtools v0.1.15 (72). Subsequently, the SNPs of all samples in the thinned subset were converted into fasta sequences using vcf2phylip v2.8 (74). The resulting sequences were then used to infer a neighbor-joining (NJ) tree by phylip v3.698 (75). An mt NJ tree was also constructed using the same method. The tree was visualized using Interactive Tree Of Life (iTOL) (http://itol.embl.de). A mt haplotype network of genotype G1, excluding G3, was generated using the median-joining network method in PopART v1.7 (76). PCA of the thinned subset was performed using PLINK v1.9 (77), calculating the top eight principal components for all samples as well as for each population. Admixture analysis of the thinned subset was performed using the block relaxation algorithm implemented in the ADMIXTURE v1.3.0 (78), with *K* values (number of hypothetical ancestral populations) ranging from 1 to 10.

Based on the whole-genome SNP data set in 2 kb sliding non-overlapping windows, VCFtools v0.1.15 (72) was employed to calculate the nucleotide diversity (π) for all samples and each population, as well as the relative divergence index (*F*_ST_) for pairwise populations. The absolute difference index (*D*_xy_) between populations was calculated by using pixy (79). Prior to calculating genome-wide mean values and their 95% CI for each index, the negative and null values of windows were corrected to zero. Linkage disequilibrium decay was assessed using popLDdecay v3.42 (80), which calculates the genotype correlation coefficient (*r*^2^) for pairs of unphased SNPs at a maximum distance range of 300 kb. The FastEPRR v2.0 (81) was used to calculate the genome-wide recombination rate along non-overlapping 50 kb windows for each population. The *F* value for heterozygosity, compared with Hardy–Weinberg expectations, was calculated for all samples using VCFtools v0.1.15 (72), which was also used to split the variant call format (VCF) data set into individual sample VCF files and calculate the number of heterozygous bases in each sample. Additionally, we utilized VCFtools v0.1.15 (72) to calculate allele frequency for all SNPs and identified those with a frequency below 5% as Low-MAF sites. Finally, bedtools v2.26.0 (82) was employed to map and count the number of Low-MAF sites in each sample’s VCF data set. For analysis of sample ploidy and intersample mixing, bcftools 1.13 (69) was used to extract the sequencing depth information for the alternate and reference alleles at all heterozygous sites for each sample. We then employed a custom Python script to exclude heterozygous sites in the tandem repeat regions annotated in the reference genome (28) and calculated the MAF of the heterozygous sites in the non-tandem repeat regions for each sample.

### Demographic history inference

To assess whether the mixture of ancestral components between populations in admixture analysis results from gene flow, we calculated the *D*-statistics (ABBA-BABA test) and f4-ratio for all possible combinations of the trios (P1, P2, and P3) using the Dtrios subcommand in Dsuite v0.5 (83), with the reference genome (28) as the outgroup. Subgroup grouping criteria for trios were based on the close relationship of a phylogenetic tree and the similar composition of ancestral components in the admixture analysis (grouping results were presented in Table S3). Trios in the Dtrios results with BH corrected *P*-value < 0.05 and *Z* score for *D* > 3 were considered to contribute to gene flow. The *f_b_* metric was implemented by the Fbranch subcommand with *P*-value < 0.05 and the script dtools.py in Dsuite v0.5 to correlate with the f4-ratio result (83). To validate the reliability of the grouping, each population was randomly divided into 10 subgroups, and 50 repeats of the grouping were performed for running Dtrios calculations.

The estimation of effective population size (*N_e_*) histories, based on unphased high-quality SNP data sets, was conducted using SMC++ v1.15.2, capable of simultaneously analyzing multiple samples and identifying recent demographic ﬂuctuations (84). The vcf2smc subcommand was executed to convert the bi-allelic SNP data set from the nine chromosomes into SMC++ format. We used the estimate subcommand in SMC++ with the knots parameter set to 18 to calculate the *N*_e_ histories for each population along window sizes of 10 bp, 50 bp, 200 bp, and 500 bp, respectively. Additionally, the CV subcommand was also employed to perform cross-validation and assess the *N*_e_ history trends with the default 100 bp window size and twofold. The real time was adjusted using the mutation rate μ = 2.31 × 10^−9^ per site per year, calculated by dividing the branch length by the divergence time of *E. granulosus*, as reported in Wang et al. (85). Finally, using this μ and the nucleotide diversity (π), we calculated the current *N*_*e*_ for the three populations by the formula π = 4*N_e_*μ (31).

### Detection of selection signals

To identify regions in the *E. granulosus s.s.* genome subjected to selective sweeps, we utilized both intra-population (*n*S_L_ and Tajima’s *D*) and cross-population (*F*_ST_ and XP-*n*S_L_) statistic methods based on the whole-genome SNP data set. Selscan v2.0 (86) was employed for calculating the *n*S_L_ and XP-*n*S_L_ without the need for genomic mapping. The normalization of all Selscan outputs across every chromosome was performed using the norm tool integrated into Selscan v2.0 (86). Mean *n*S_L_ and XP-*n*S_L_ scores were computed within non-overlapping 2 kb windows along each chromosome. For XP-*n*S_L_ calculations, the XJ population served as the reference population, with the QG and XZ populations as the target populations. Using VCFtools v0.1.15 (72) under the same sliding window conditions, we calculated Tajima’s *D* and *F*_ST_ between populations. Candidate selection windows were defined as the top 1% of windows in the genome with the highest ranking of each analytical value. Subsequently, the intersect subcommand in bedtools v2.26.0 (82) was employed to identify genes overlapping with these candidate selection windows, considering them as candidate genes subject to selective sweeps. For GO and KEGG enrichment analyses of identified candidate genes, the clusterProfiler package v4.0 (87) in R was utilized. After applying the BH correction, terms with *P*-values < 0.05 were considered significantly enriched. The LDBlockShow v1.39 (88) was used to generate LD heatmaps for the local genomic region and the focused gene. The rehh package v3.2.2 (89) in R was employed to plot haplotype decay for the sites under linked selection. Finally, to ascertain whether the regions under linked selection were driven by balancing selection, we utilized the BalLeRMix v2.2 (90) to calculate the *B*_2_ statistics for the non-overlapping 2 kb windows based on the minor allele frequency spectrum with default parameters. Regions with the top 1% *B*_2_ values (576.20) were identified as potentially subjected to balancing selection.

## Data Availability

Sequencing data after basic quality control have been deposited into CNGB Sequence Archive (CNSA) of CNGBdb (https://db.cngb.org/cnsa/) under project accession CNP0002581. The code used for data analysis is available at https://github.com/WYD-510/Code-of-Echinococcus-granulosus-sensu-stricto-population-study.

## AFTER EPUB

*[This article was published on 10 April 2025 with errors in the affiliations. The affiliations were corrected in the current version, posted on 23 April 2025.]*

## SUPPLEMENTAL MATERIAL

Supplemental Figures mbio.03256-24-s0001.docx Figures S1–S20.

Supplemental Tables mbio.03256-24-s0002.xlsx Tables S1–S13.

ASM does not own the copyrights to Supplemental Material that may be linked to, or accessed through, an article. The authors have granted ASM a non-exclusive, world-wide license to publish the Supplemental Material files. Please contact the corresponding author directly for reuse.
