# Supplementary material for: Whole-genomic comparison reveals complex population dynamics and parasitic adaptation of Echinococcus granulosus sensu stricto
Source: mBio. 2025 Apr 10;16(5):e03256-24. doi: 10.1128/mbio.03256-24 (PMC12077126; doi:10.1128/mbio.03256-24)
Supplement: Supplemental Figures — Figures S1–S20. [file mbio.03256-24-s0001.docx]

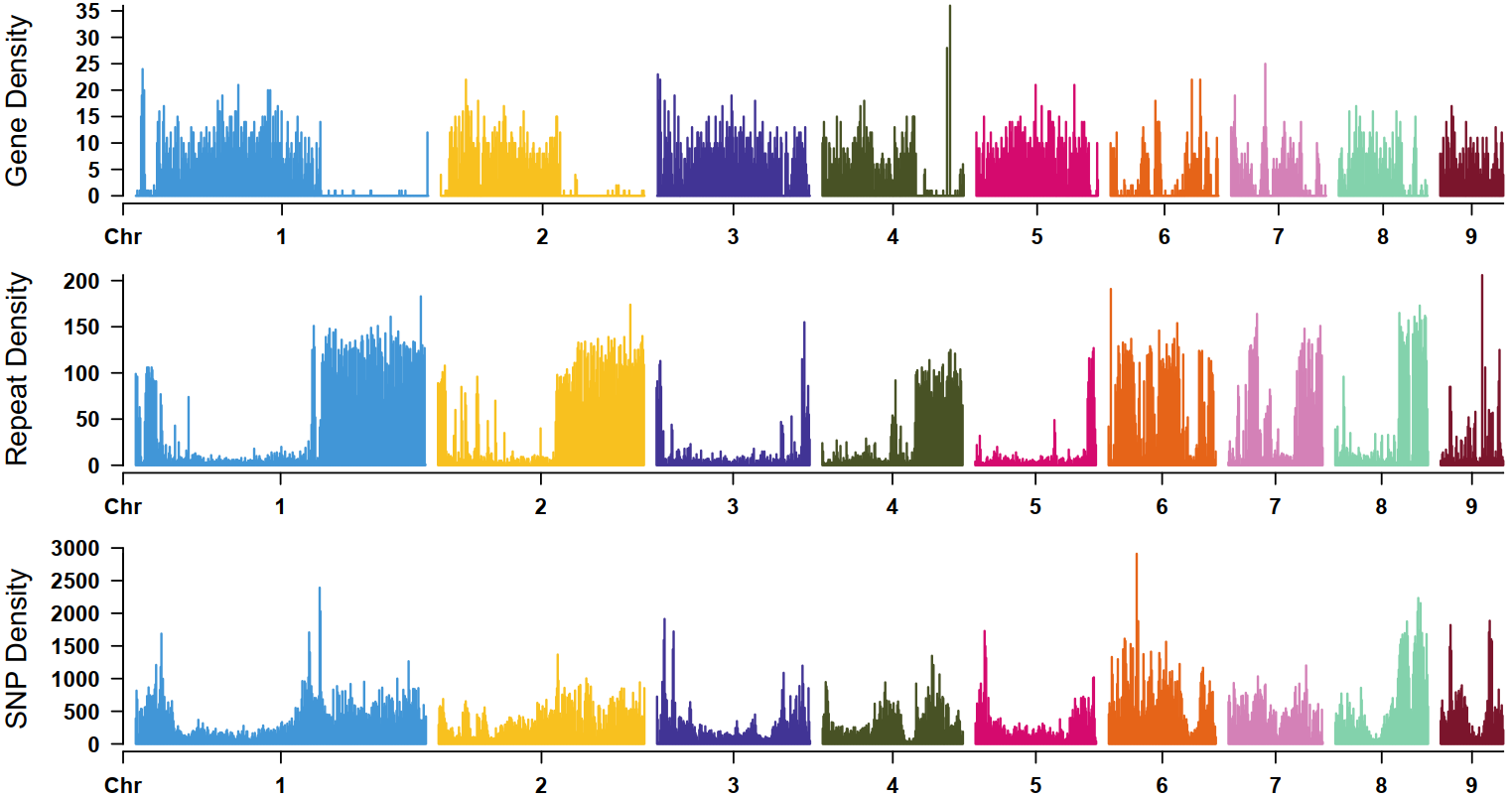


**Figure S1. Manhattan plots depicting the density distribution of genes, repeat sequences and SNPs along 100kb windows.** The density statistics of genes and repeat sequences were based on the reference genome published by Korhonen et al. (S1), and the density statistics of SNPs were based on the total SNPs dataset in this study. Chr1 to Chr9 correspond to the chromosome IDs of GenBank listed in Table S2.


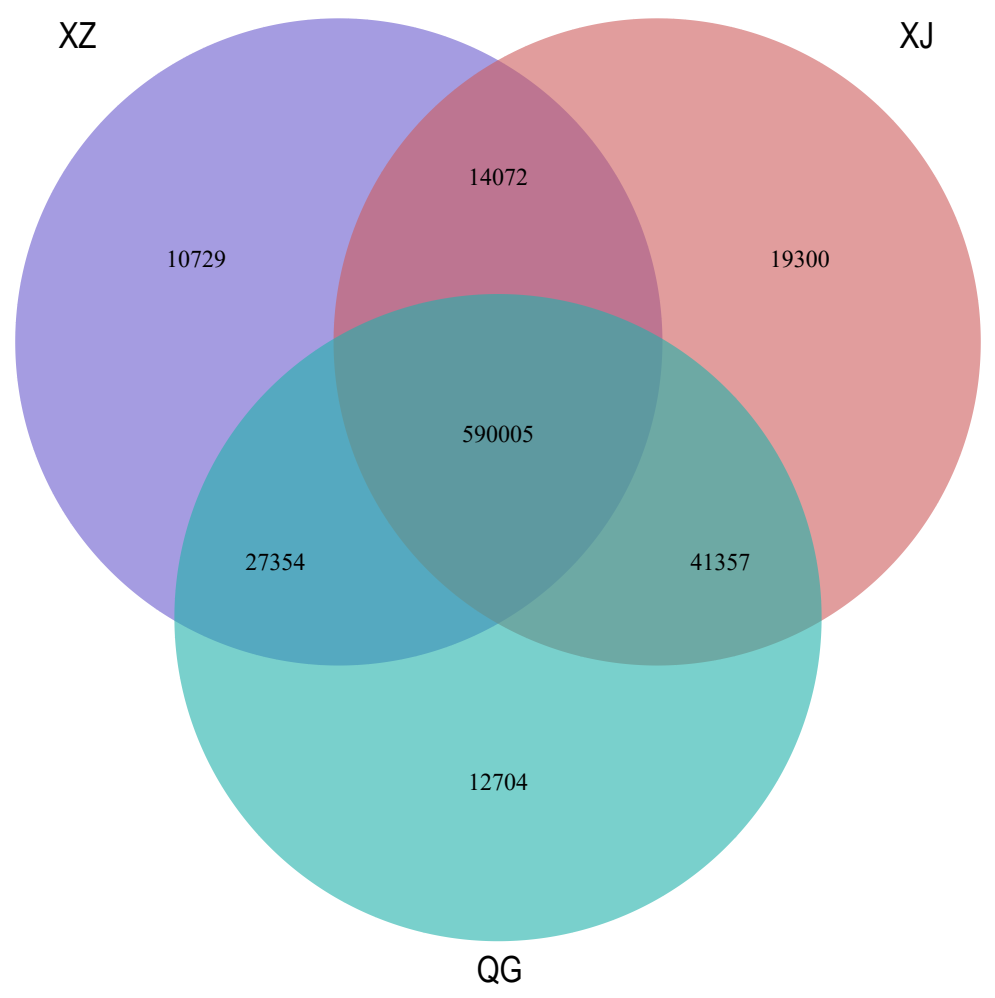


**Figure S2. Venn diagram of SNPs identified in XJ, QG and XZ populations.**


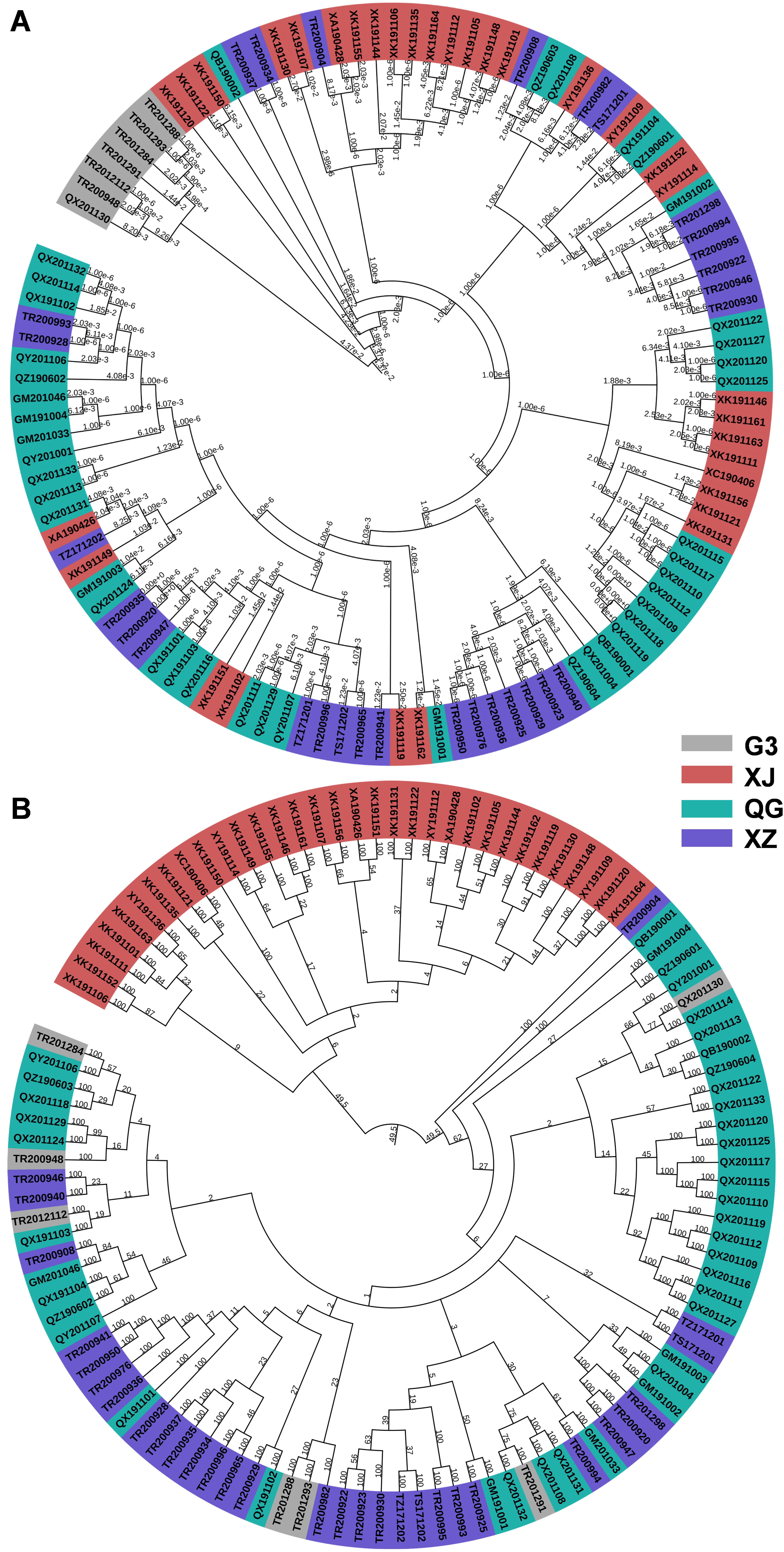


**Figure S3. The neighbor-joining (NJ) phylogenetic trees for mitochondrial and nuclear genomes, labeled with sample IDs and branch lengths.** (A) The phylogenetic tree corresponding to Fig. 1B; (B) The phylogenetic tree corresponding to Fig. 1D.


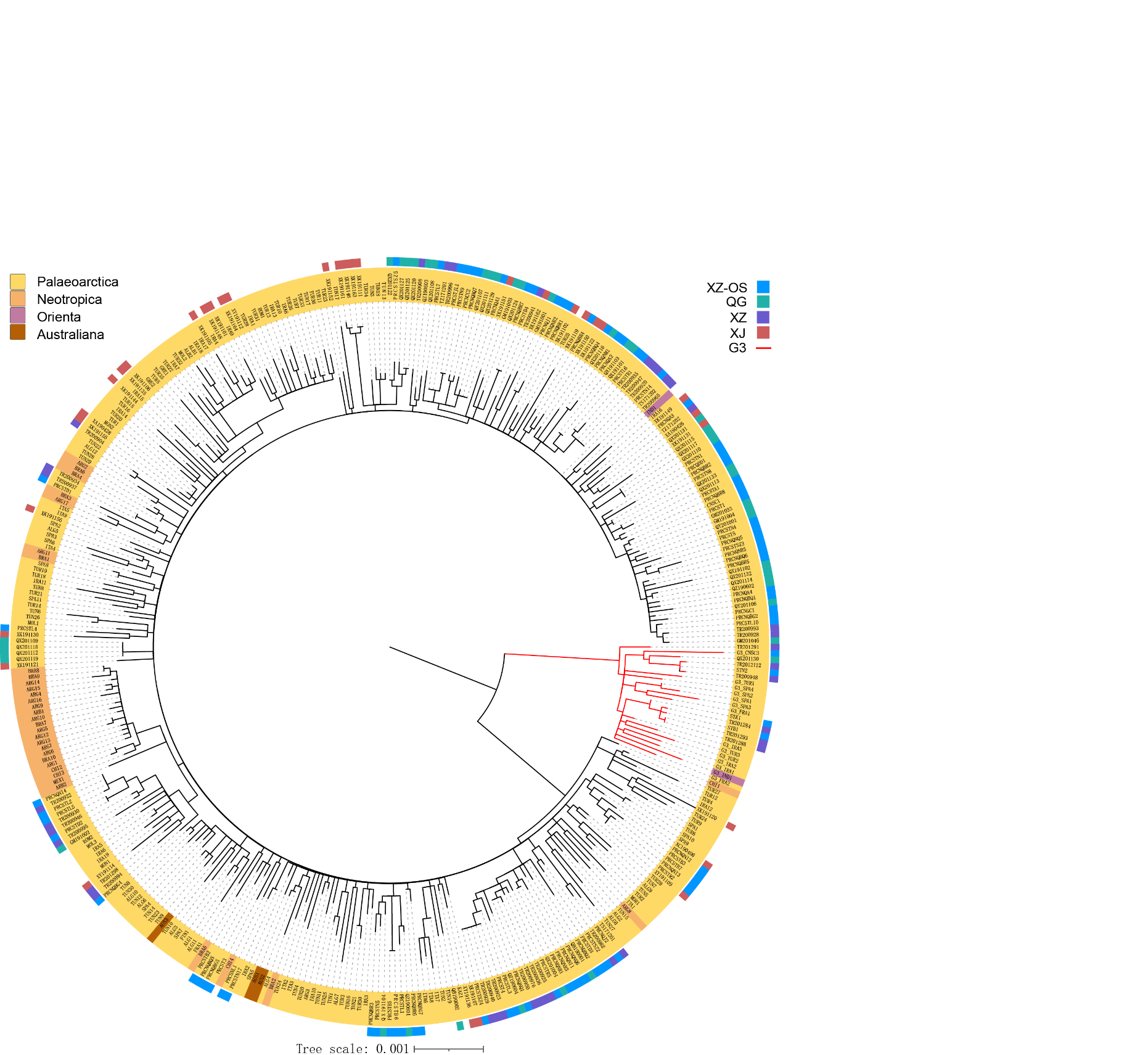


**Figure S4. Phylogenetic tree of 376 globally distributed *Echinococcus granulosus* s.s. mitochondrial genomes.** The tree was constructed with maximum likelihood (ML) inference using IQ-TREE v2.1.4 with ultrafast bootstrap 1,000 replicates, where the best-ﬁt models were automatically selected by ModelFinder and the best number of threads were also selected under AUTO option (S2). The background colors of the sample IDs represent the faunal region of the samples located in. The outer circles with different colored bars depict samples from different regions in China, with XZ-OS representing near-complete mitogenome sequences from Zhao et al. (S3). The other sample IDs in the figure are derived from near-complete mitogenome sequences of *E. granulosus* s.s. published by Wang et al. (S4) and Kinkar et al. (S5).


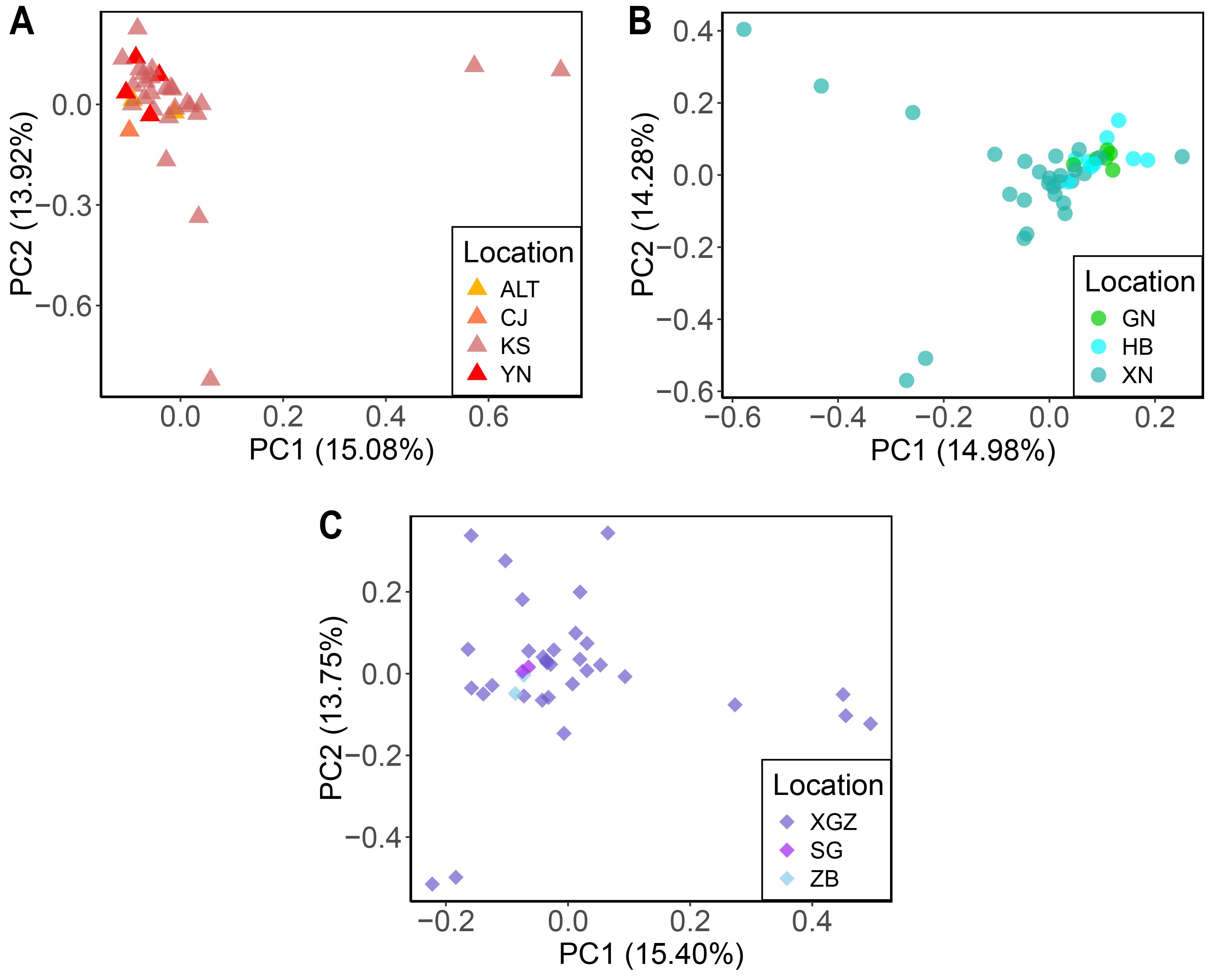
**Figure S5. The within-population PCA of XJ (A), QG (B), and XZ (C) populations, respectively.** The abbreviations of different sampling locations correspond to those shown in Fig. 1A.


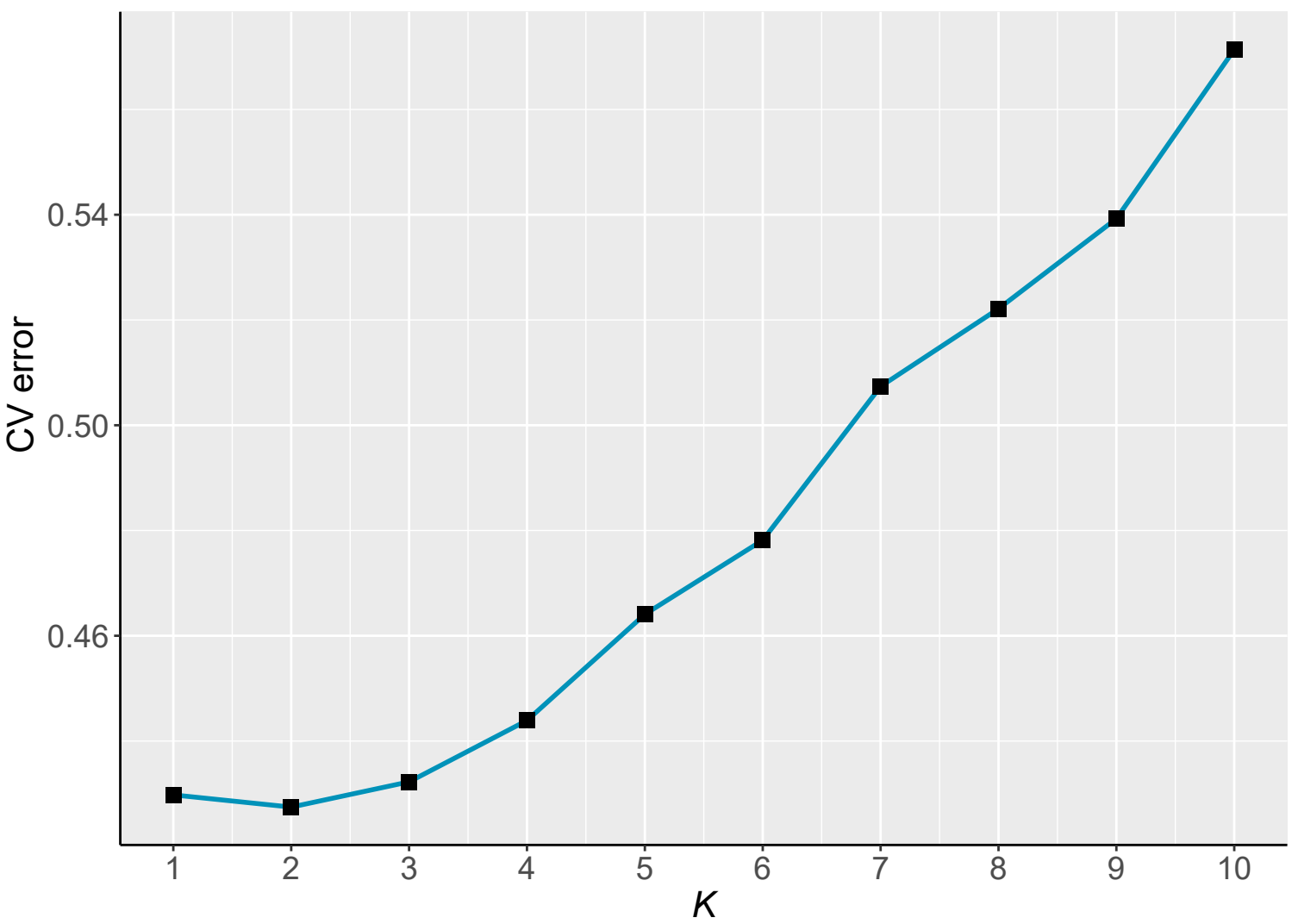


**Figure S6. Cross-validation plot for the ADMIXTURE analyses. *K* ranges from 1 to 10.**


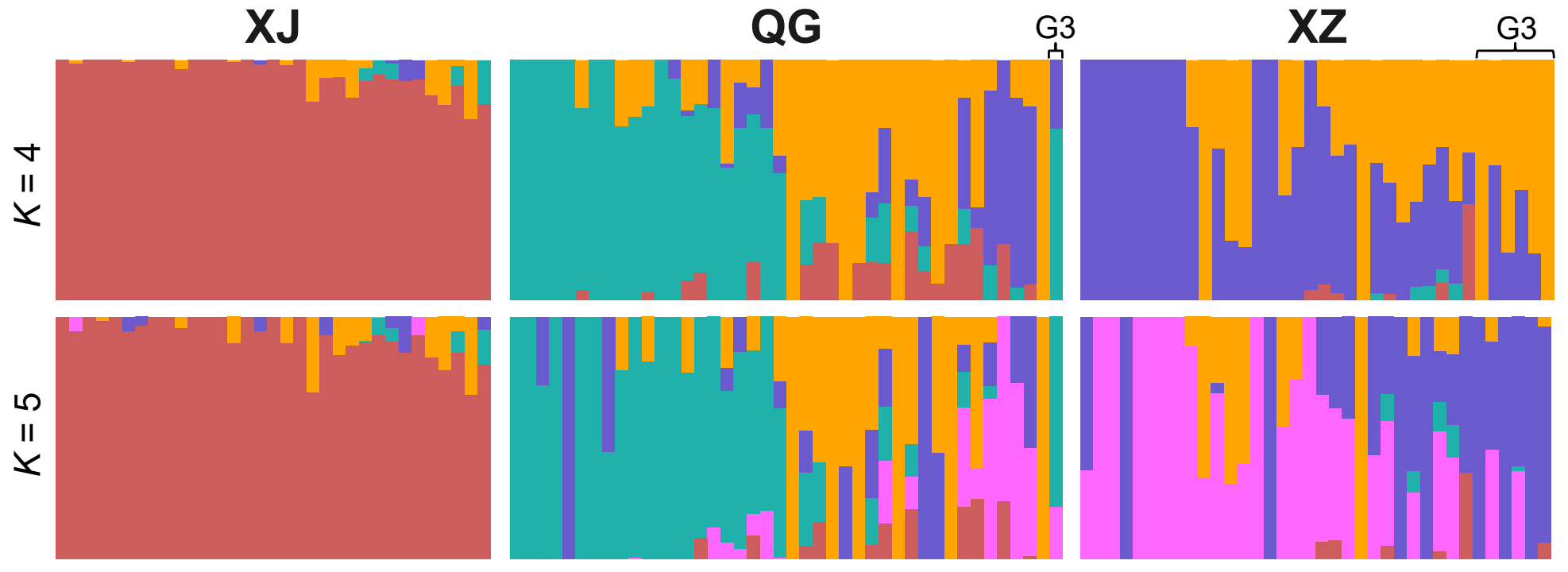


**Figure S7.** **The genomic ancestry composition of the three populations for *K* = 4 and 5 in ADMIXTURE analysis.**

**
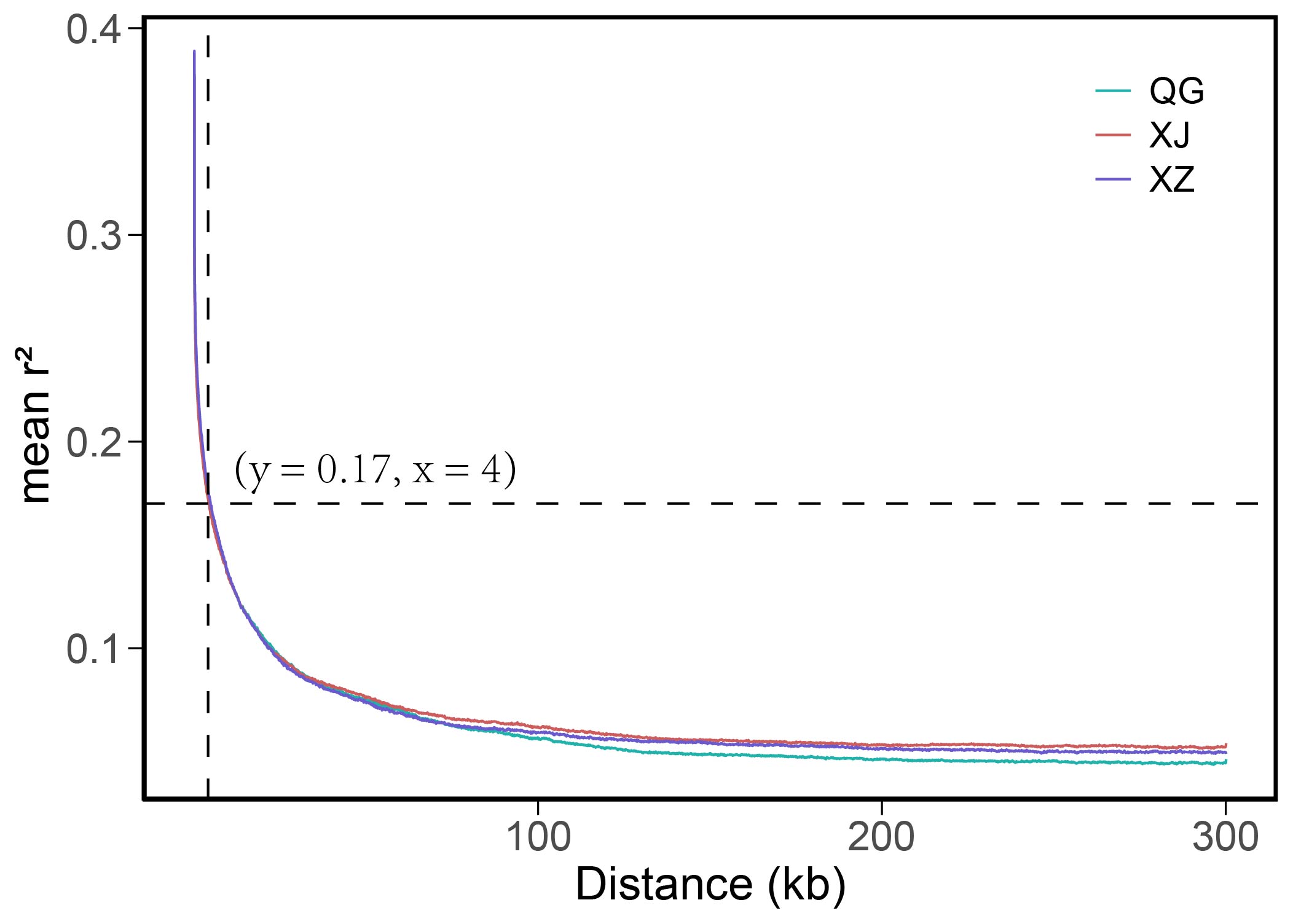
**

**Figure S8. Linkage disequilibrium (LD) decay in different populations.** The dashed line in the figure indicates the genetic distance at which the mean *r²* value for the three populations decays to half of its initial value (0.17), corresponding to a distance of 4 kb.

**
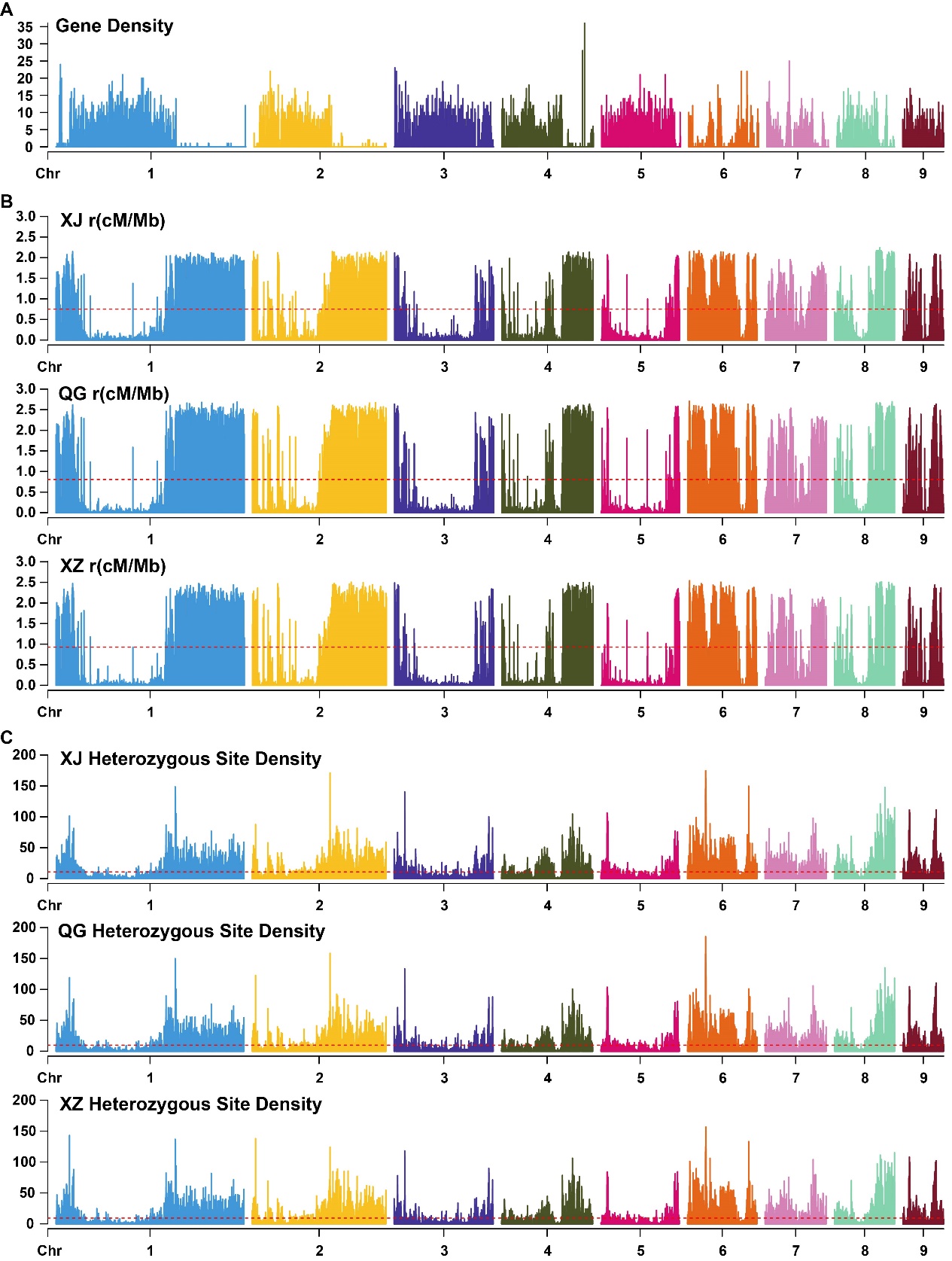
Figure S9. Manhattan plots showing gene density distribution in 100 kb windows along the genome of *E. granulosus* s.s. (A), recombination rate (r) in 50 kb windows (B) and heterozygous base density in 10 kb windows (C) for different populations.** The red dashed lines in panel B represent horizontal line at mean value shown in Table 2. The red dashed lines in panel C represent the horizontal lines corresponding to the average number of heterozygous sites in 10 kb windows for the XJ, QG, and XZ populations, which are 10.84, 9.88 and 9.37, respectively.


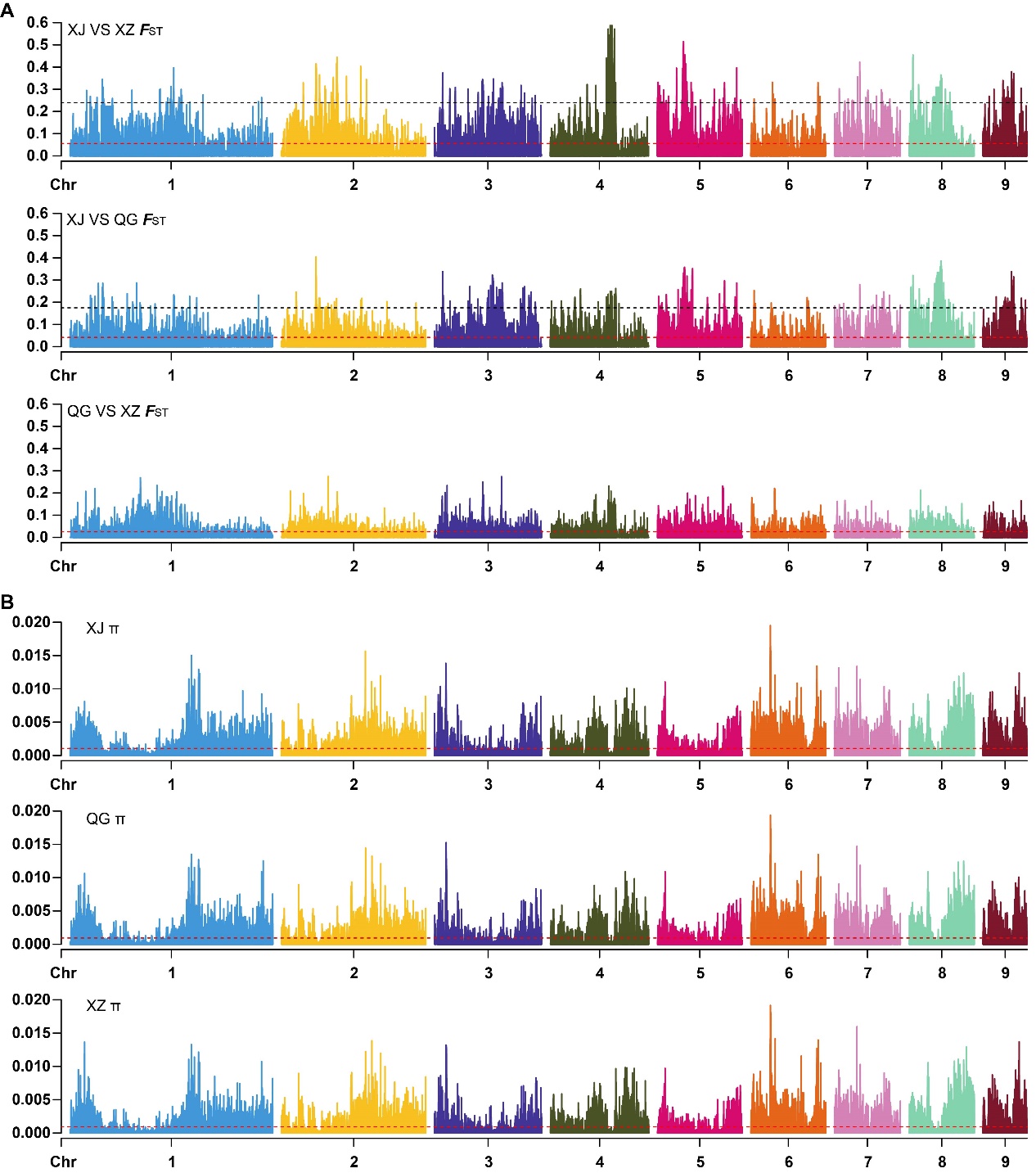
**Figure S10. Manhattan plots of the cross-population *F*_ST_ (A) and the intra-population π (B)** **along non-overlapping 2 kb windows.** All the red dashed lines represent horizontal line at mean value shown in Table 2. The black dashed line in *F*_ST_ Manhattan plot represents the threshold of top 1% value.


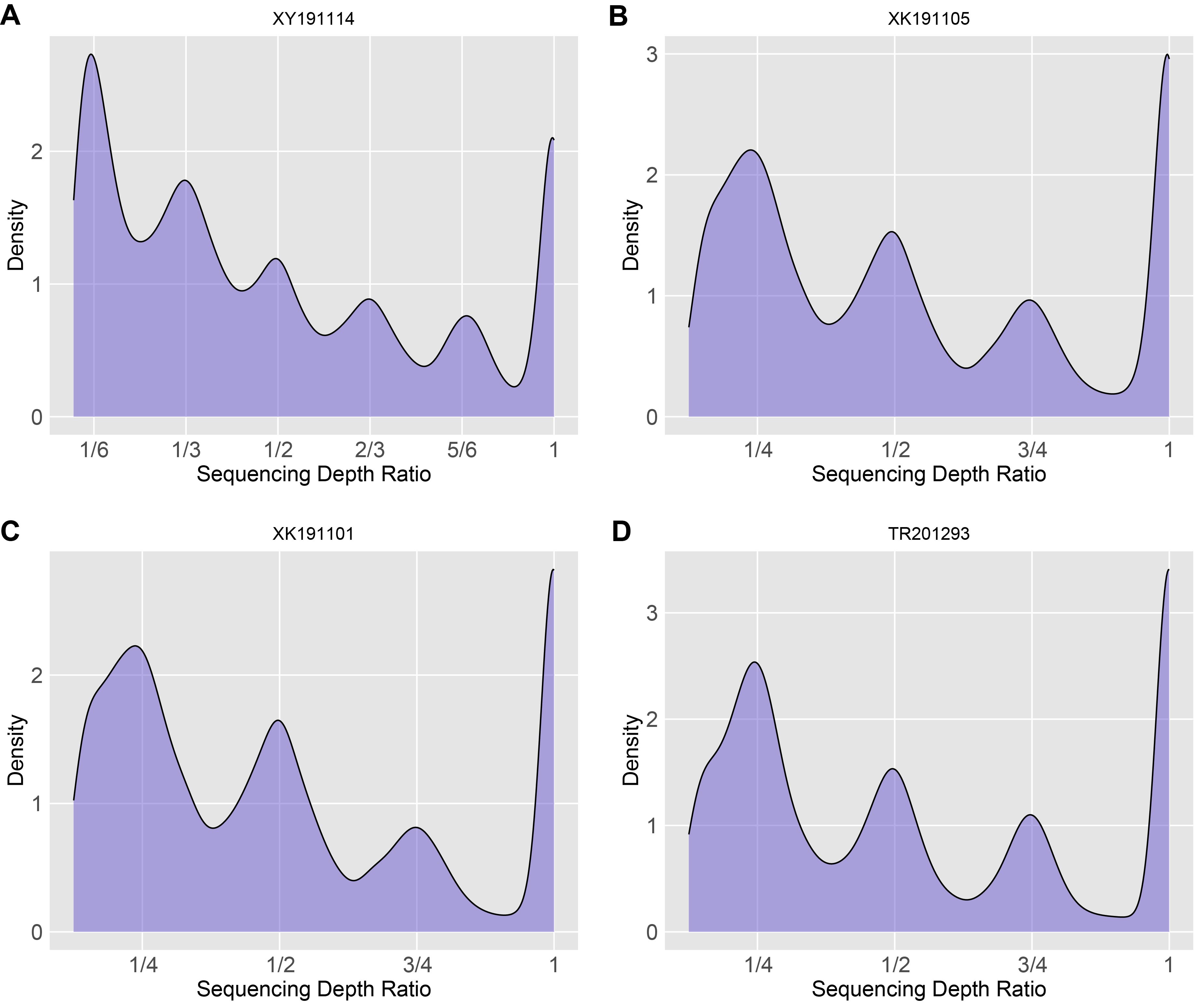


**Figure S11.** **Density plots of the ratio between the sequencing depth of alternate allele sites and the total sequencing depth at the corresponding sites (alternate allele frequency) for the four polyploid samples.** The six peaks at 1/6, 1/3, 1/2, 2/3, 5/6 and 1 of the hexaploid sample XY191114 (A) represent the allelic configurations AAAAAB, AAAABB, AAABBB, AABBBB, ABBBBB and BBBBBB, respectively. The four peaks at 1/4, 1/2, 3/4 and 1 of the tetraploid sample XK191105 (B), XK191101 (C) and TR201293 (D) represent the allelic configurations AAAB, AABB, ABBB and BBBB, respectively.


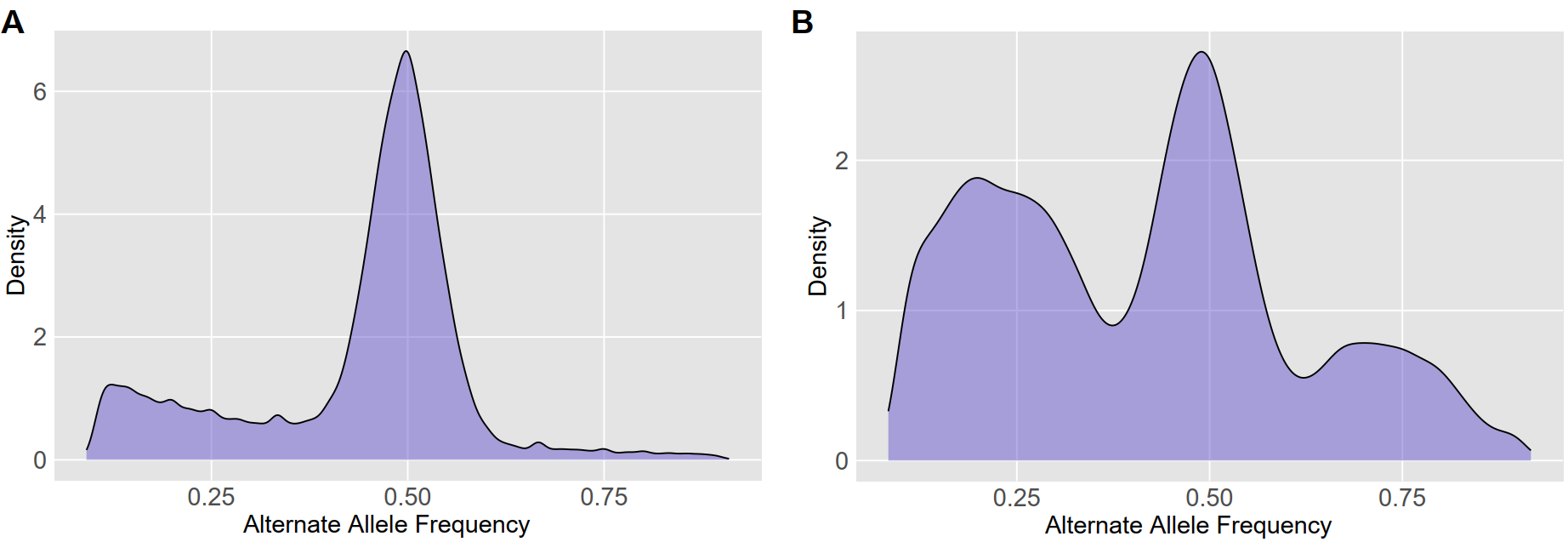


**Figure S12.** **Density plot of alternate allele frequencies for heterozygous sites in non-tandem repeat regions.** (A) represents pure diploid samples; (B) represents mixed diploid samples, where the unusually high low-frequency and high-frequency peaks correspond to the variation frequencies of the minor component samples and major component samples, respectively.


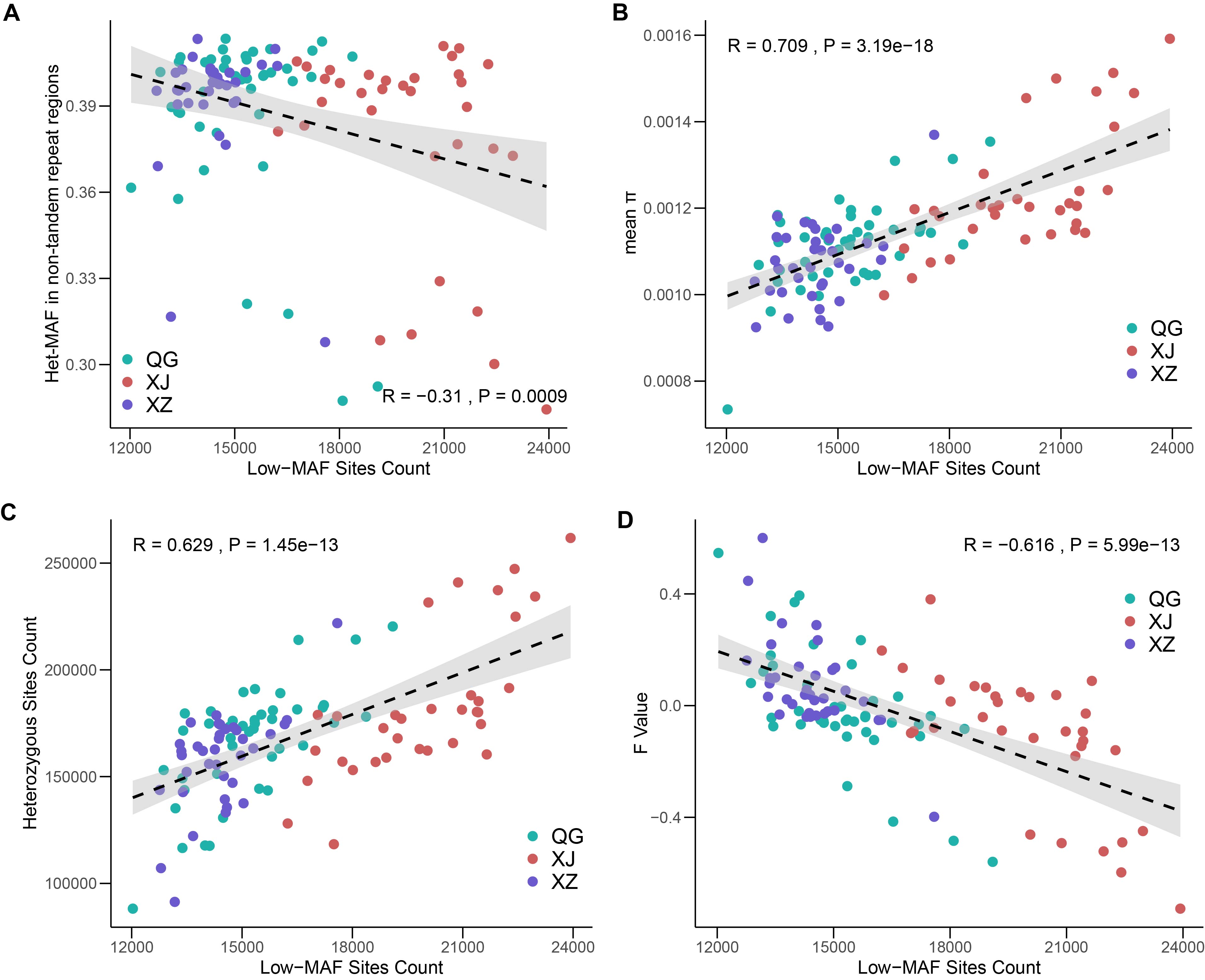


**Figure S13. Correlation analysis between each sample’s** **Low-MAF sites count and their Het-MAF in non-tandem repeat regions (A), nucleotide diversity π (B), heterozygous site count (C) and *F* value (D).**


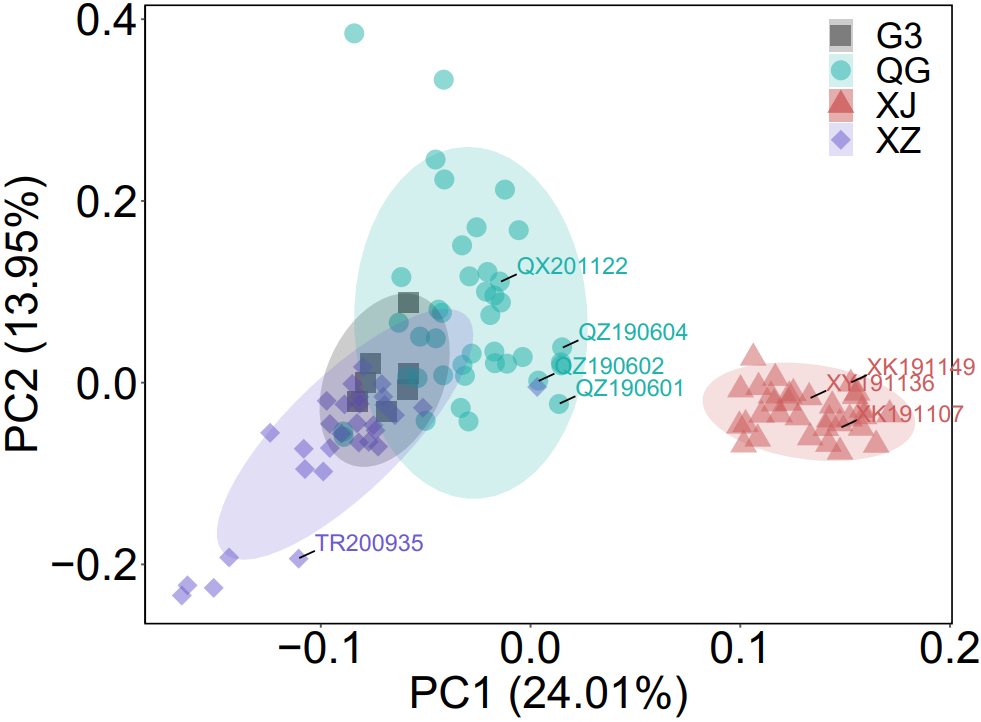


**Figure S14. PCA plot of the nuclear genome with mixed sample IDs marked, corresponding to Fig. 1E.**


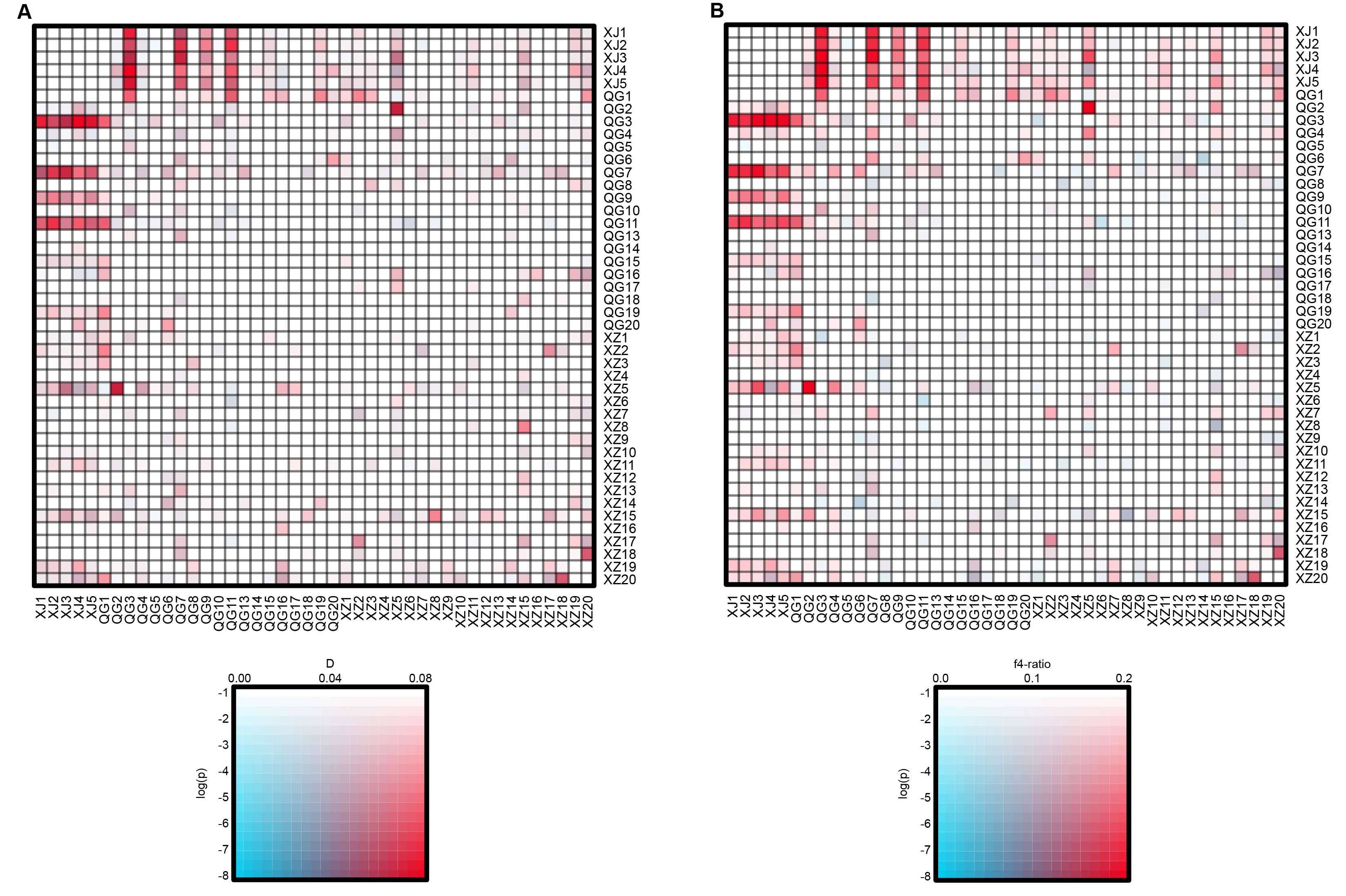


**Figure S15. Heatmaps depicting the most significant *D*-statistics (A) and f4-ratio (B) values found among all possible P1 with fixed P2 and P3 in the trios that contribute to gene flow.** The samples belonging to subgroups in the trios were detailed in supplementary table S4. Trios in the Dtrios results with Benjamini-Hochberg (BH) corrected *p*-value < 0.05 served as the data source for generating these two heatmaps using the scripts plot_d.rb and plot_f4ratio.rb (<https://github.com/millanek/tutorials/tree/master/analysis_of_introgression_with_snp_data>).


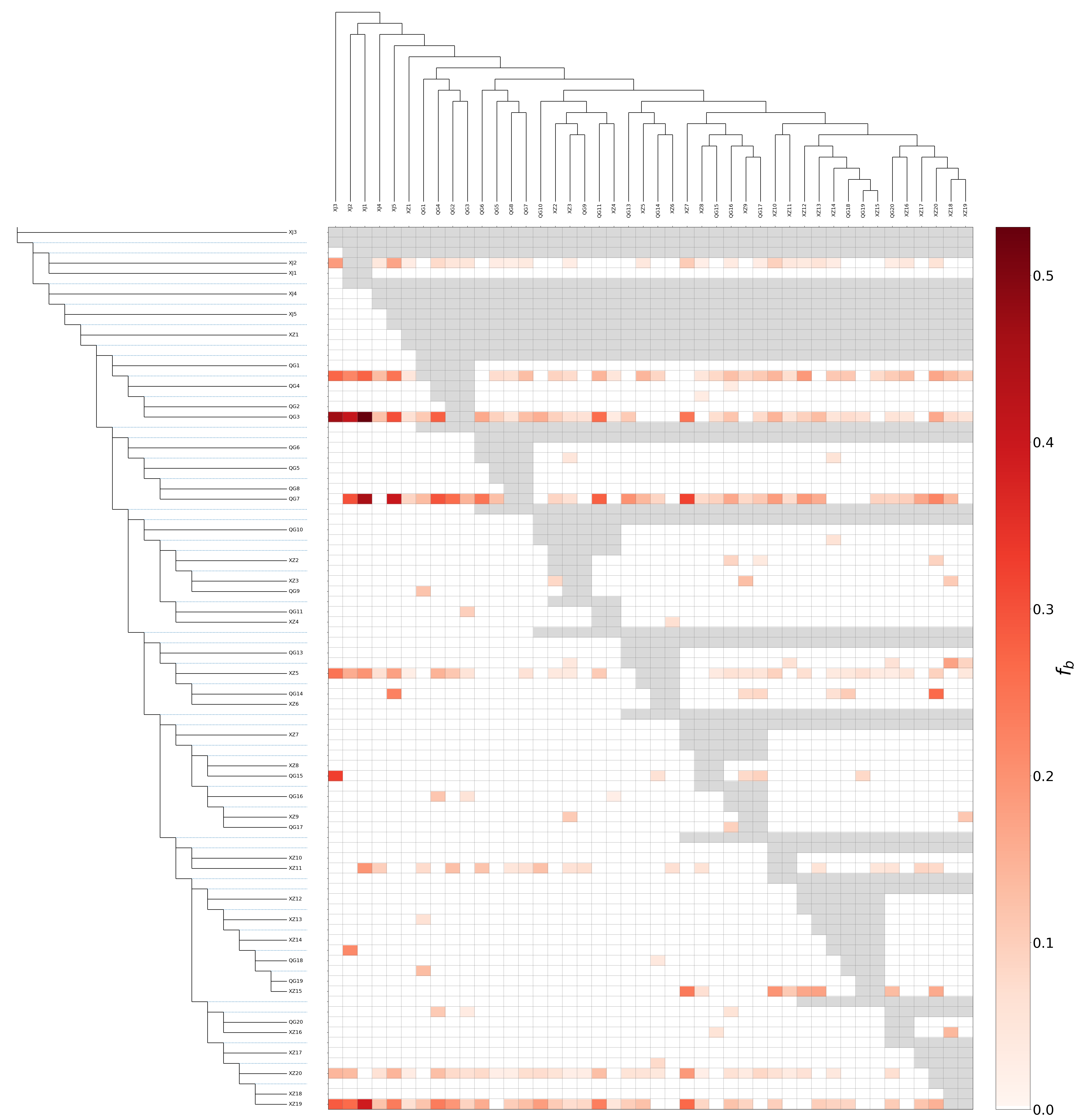


**Figure S16. The f-branch (*****f_b_*) metric for identification of possible internal branches and gene flows between subgroups.** The branch-specific statistic *f_b_* measures excess of alleles sharing between the branch of the tree on the *y* axis with the clade on the *x* axis. The file with the suffix _tree.txt generated by running the Dtrios in Dsuite program served as the data source. The merged phylogenetic tree according to Table S3 was used as a basis for the branch statistic. The grey data points in the matrix correspond to tests that are not consistent with the phylogeny.


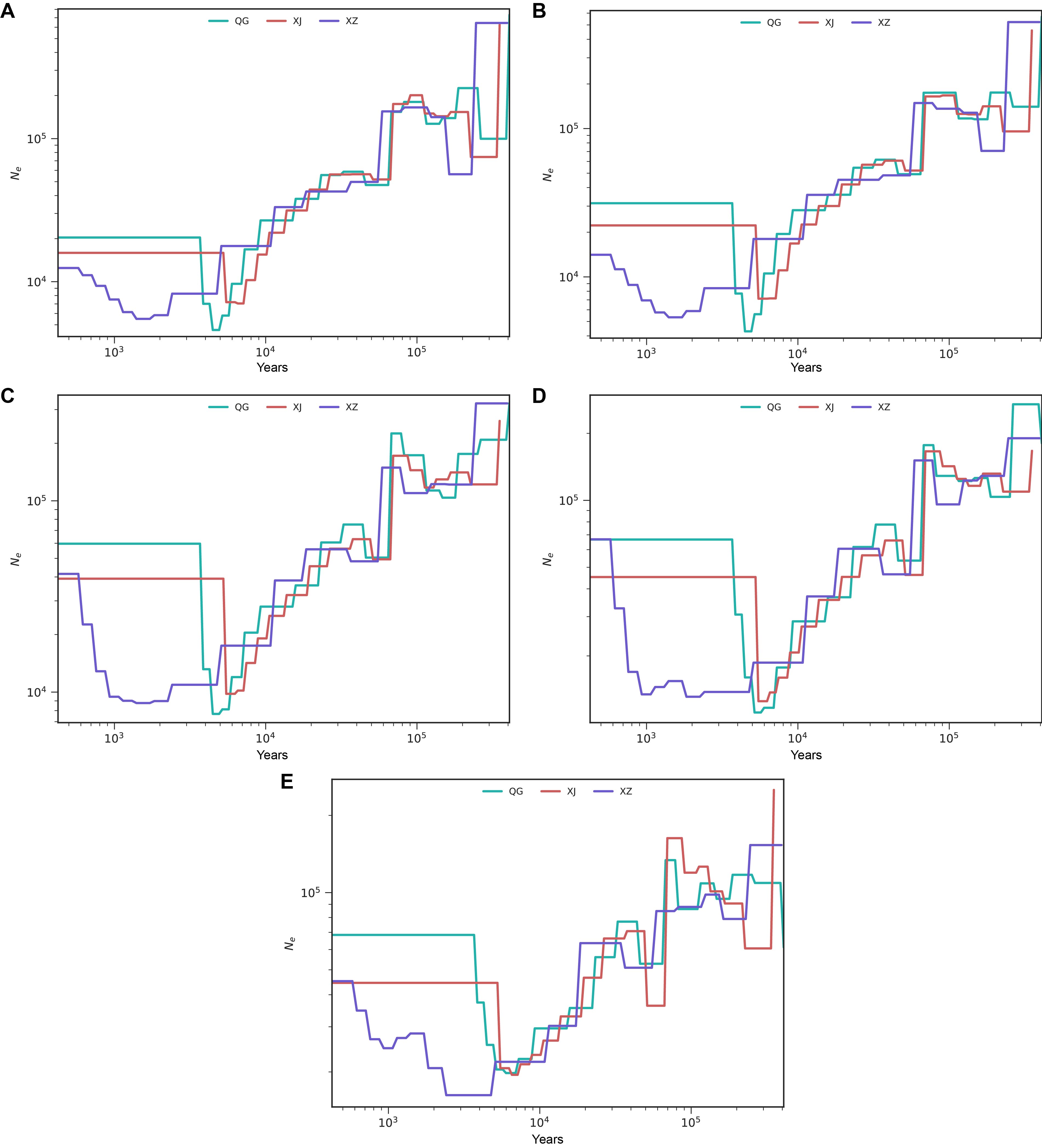


**Figure S17. Inference of historical changes in effective population size (*N_e_*) for the three populations using SMC++ with window sizes of 10bp (A), 50bp (B), 200bp (C), 500bp (D), and 1000bp (E).**


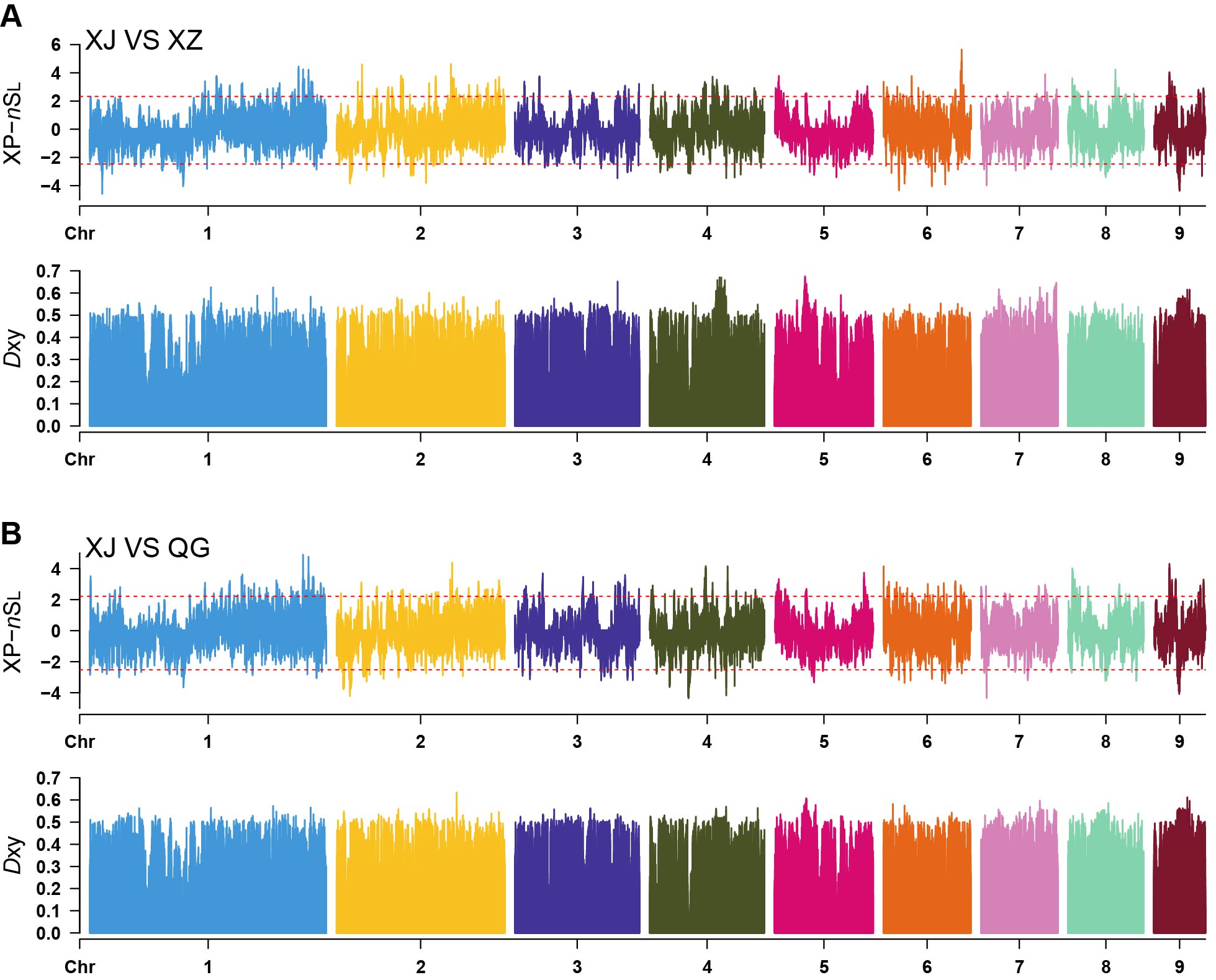


**Figure S18.** **Manhattan plots of the cross-population statistic methods (****XP-*n*S_L_ and** ***D*_xy_) calculated along non-overlapping 2 kb windows between XJ and XZ (A), as well as between XJ and QG (B).** The red dashed line of positive and negative values in XP-*n*S_L_ manhattan plot represents the threshold of top 1% value for the selection direction in the target and reference populations, respectively.


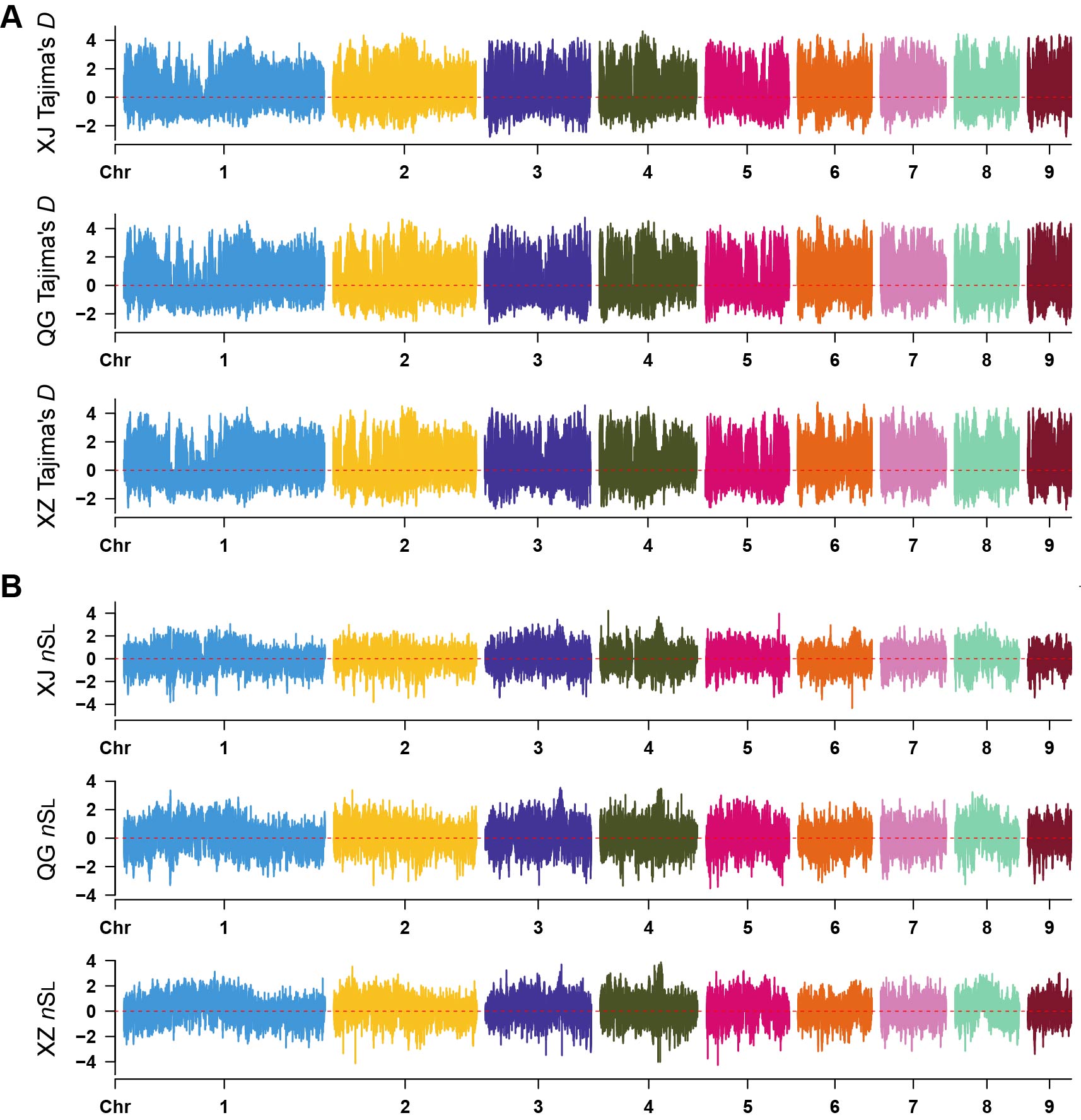


**Figure S19. Manhattan** **plots of the** **intra-population statistic methods, Tajimas’ *D* (A) and *n*S_L_ (B), calculated along non-overlapping 2 kb windows for each of the three populations.** The red dashed lines represent horizontal line at zero value.

**
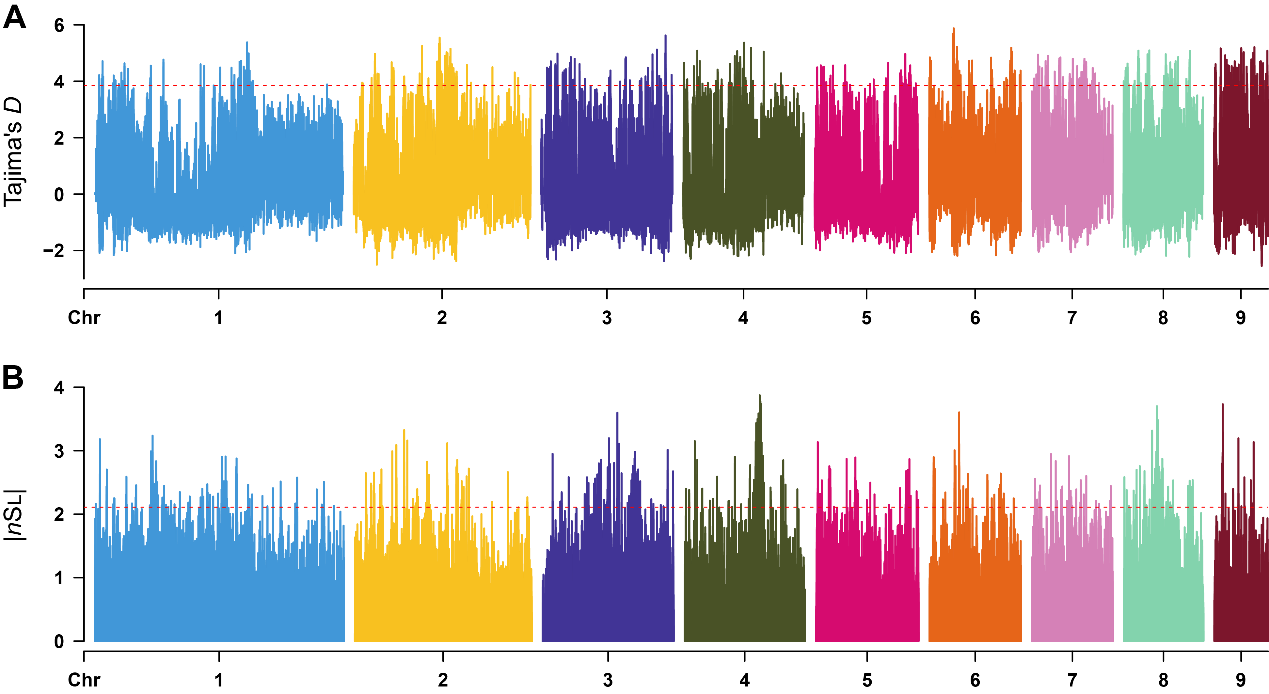
**

**Figure S20.** **Manhattan plots of intra-population selection signals for all samples in this study.** (A) Manhattan plot of Tajima’s *D* value calculated along 2 kb non-overlapping windows. (B) Manhattan plot of the median |*n*S_L_| values calculated along 2 kb non-overlapping windows. The red dashed line represents the threshold of the top 1% absolute value.

**Supplemental References:**

S1. Korhonen PK, Kinkar L, Young ND, Cai H, Lightowlers MW, Gauci C, et al. 2022. Chromosome-scale *Echinococcus granulosus* (genotype G1) genome reveals the Eg95 gene family and conservation of the EG95-vaccine molecule. Commun Biol 5: 199. <http://doi.org/10.1038/s42003-022-03125-1>.

S2. Nguyen LT, Schmidt HA, von Haeseler A, Minh BQ. 2015. IQ-TREE: a fast and effective stochastic algorithm for estimating maximum-likelihood phylogenies. Molecular Biology and Evolution 32:268-274. <https://doi.org/10.1093/molbev/msu300>.

S3. Zhao Y, Gesang D, Wan L, Li J, Qiangba G, Danzeng W, Basang Z, Renzhen N, Yin J, Gongsang Q, Cai H, Pang H, Wang D, Asan, Zhang Q, Li J, Chen W. 2022. *Echinococcus* spp. and genotypes infecting humans in Tibet Autonomous Region of China: a molecular investigation with near-complete/complete mitochondrial sequences. Parasites & Vectors 15:75. <https://doi.org/10.1186/s13071-022-05199-6>.

S4. Wang N, Xie Y, Liu T, Zhong X, Wang J, Hu D, Wang S, Gu X, Peng X, Yang G. 2016. The complete mitochondrial genome of G3 genotype of *Echinococcus granulosus* (Cestoda: Taeniidae). Mitochondrial DNA. Part A, DNA Mapping, Sequencing, and Analysis. 27:1701-1702. <https://doi.org/10.3109/19401736.2014.961129>.

S5. Kinkar L, Laurimae T, Acosta-Jamett G, Andresiuk V, Balkaya I, Casulli A, Gasser RB, van der Giessen J, Gonzalez LM, Haag KL, Zait H, Irshadullah M, Jabbar A, Jenkins DJ, Kia EB, Manfredi MT, Mirhendi H, M'Rad S, Rostami-Nejad M, Oudni-M'Rad M, Pierangeli NB, Ponce-Gordo F, Rehbein S, Sharbatkhori M, Simsek S, Soriano SV, Sprong H, Snabel V, Umhang G, Varcasia A, Saarma U. 2018. Global phylogeography and genetic diversity of the zoonotic tapeworm *Echinococcus granulosus* sensu stricto genotype G1. International Journal for Parasitology 48:729-742. <https://doi.org/10.1016/j.ijpara.2018.03.006>.
